# Ultrasound-assisted transition-metal-free catalysis: a sustainable route towards the synthesis of bioactive heterocycles

**DOI:** 10.1039/d2ra02063g

**Published:** 2022-05-11

**Authors:** Biplob Borah, L. Raju Chowhan

**Affiliations:** School of Applied Material Sciences, Centre for Applied Chemistry, Central University of Gujarat Gandhinagar-382030 India rchowhan@cug.ac.in

## Abstract

Heterocycles of synthetic and natural origin are a well-established class of compounds representing a broad range of organic molecules that constitute over 60% of drugs and agrochemicals in the market or research pipeline. Considering the vast abundance of these structural motifs, the development of chemical processes providing easy access to novel complex target molecules by introducing environmentally benign conditions with the main focus on improving the cost-effectiveness of the chemical transformation is highly demanding and challenging. Accordingly, sonochemistry appears to be an excellent alternative and a highly feasible environmentally benign energy input that has recently received considerable and steadily increasing interest in organic synthesis. However, the involvement of transition-metal-catalyst(s) in a chemical process often triggers an unintended impact on the greenness or sustainability of the transformation. Consequently, enormous efforts have been devoted to developing metal-free routes for assembling various heterocycles of medicinal interest, particularly under ultrasound irradiation. The present review article aims to demonstrate a brief overview of the current progress accomplished in the ultrasound-assisted synthesis of pharmaceutically relevant diverse heterocycles using transition-metal-free catalysis.

## Abstract 2

The review summarizes the recent advances for the synthesis of bioactive heterocyclic compounds by ultrasound-assisted transition-metal-free catalysis.

## Graphical abstract

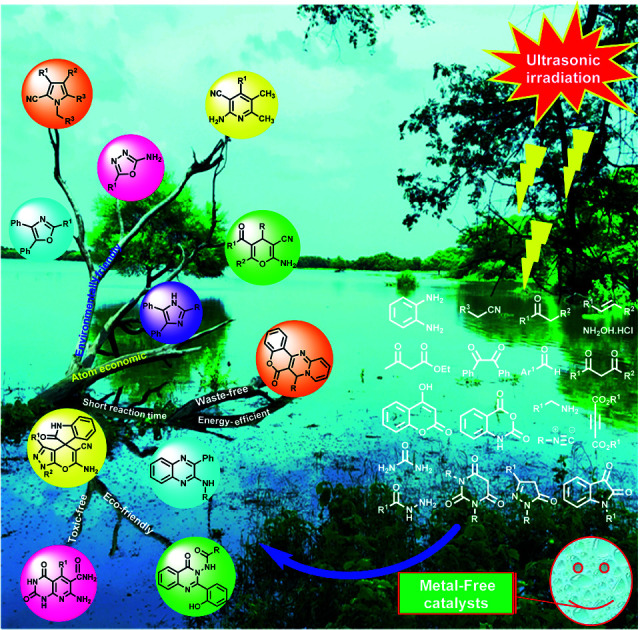

## 1. Introduction

**Fig. 1:**
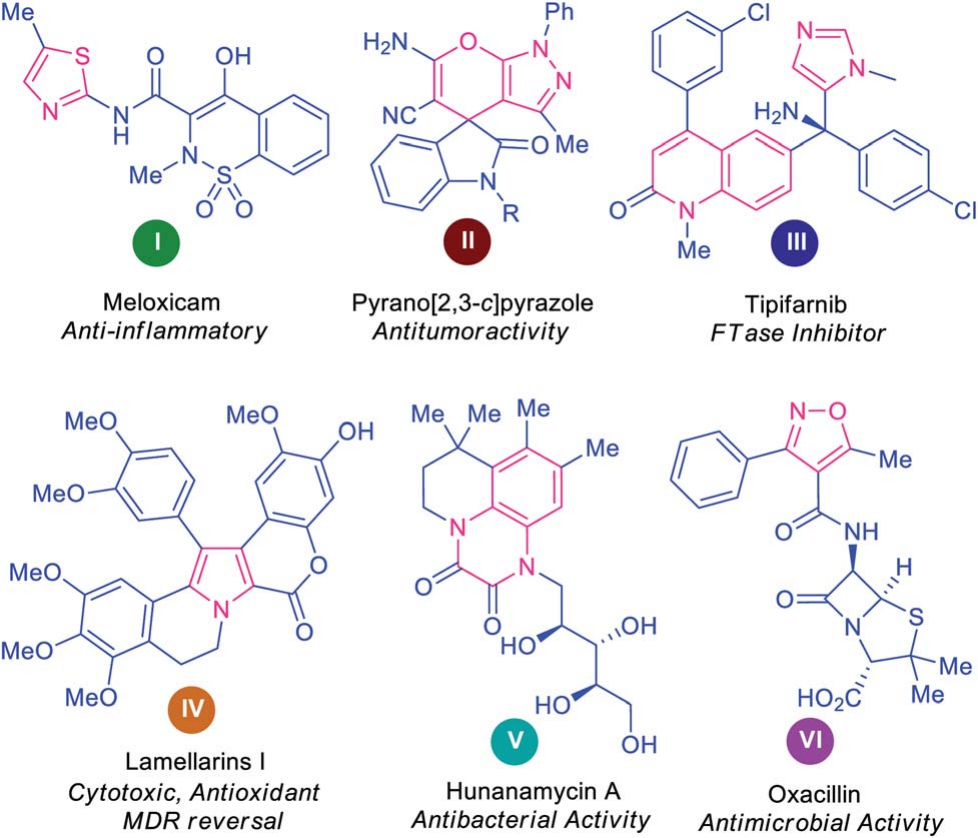
Some examples of bioactive natural and synthetic heterocyclic compounds.

Over 60% of the organic compounds produced by various chemical industries in the form of fine chemicals, drugs, pharmaceutical targets, and agrochemicals consist of heterocycles as key active ingredients.^1^ These structural motifs are not only encountered in the architecture of numerous drug candidates^2^ but are also well distributed as precursors of numerous natural products^3,4^ and optoelectronic materials.^5^ Indeed, heterocyclic compounds hold a pivotal position in the area of medicinal chemistry on account of their remarkable therapeutic potential, including anticancer, antimicrobial, antidiabetic, antioxidant, antituberculosis, antimalarial, anti-HIV, anti-hyperglycemic, and anti-dyslipidemic activity.^6–13^ Some representative examples of naturally occurring molecules and synthetic heterocycles with potential pharmacological applications are presented in Fig. 1.

Notwithstanding the tremendous pharmaceutical application, a diversified structural scaffold comprising a heterocyclic core has been demonstrated to be a crucial element in multiple materials science sectors. For instance, organic light-emitting diodes (OLEDs),^14^ organic optoelectronic devices,^15^ organic solar cells,^16^ organic semiconductors,^17^ potential fluorescent materials,^18^ electroluminescent components,^19^ and dye-sensitized solar cells are some of the promising applications of heterocyclic compounds in materials science, among others.^20^

Considering the wide abundance of heterocyclic compounds and their well-established potentiality in synthetic, organic and medicinal chemistry and materials science, the development of efficient synthetic routes providing easy access to these structural features has remained a continuous challenge for chemists in academia and industry. However, with the ever-increasing public awareness about protecting the living environment from chemical pollution associated with the chemical process, the fields of synthetic, organic and medicinal chemistry have witnessed tremendous growth in the last two decades towards making the chemical process environmentally benign and more sustainable. In this pursuit, the use of ultrasound irradiation as a highly feasible and alternative eco-friendly activation method in organic synthesis has increased substantially owing to its capability to reduce reaction times and enhance product yields by avoiding higher energy requirements.^21,22^ The ability to accomplish or promote chemical reactions using sound waves triggered by ultrasound can make the sonochemical-assisted synthetic method superior to the conventional method in terms of green and sustainable chemistry.^23–30^ Ultrasound irradiation produced substantial cavitation bubbles upon exposure to a reaction mixture after the pressure reached a certain threshold. These cavitation bubbles underwent violent collapse after growing up rapidly to facilitate the formation of a fine emulsion between the starting materials and elevate the local temperature of the reaction mixture to cross the reaction's activation energy.^31–34^

On the other hand, catalysts hold a significant position in organic chemistry for accelerating a diverse range of chemical transformations. Mainly, transition-metal-catalysts have found tremendous application in organic synthesis over the past decades for their successful utilization in the assembly of natural product analogs and therapeutically promising compounds featuring heterocycles as the critical template. However, their exploitation in organic synthesis sometimes somewhat affects the eco- and environmentally benign nature of the chemical transformation. Not all but sometimes transition-metal-catalyzed processes are found to be very sensitive to air and moisture, and a high cost is required for the preparation of the catalyst(s). In addition, non-commercial supporting ligands, co-catalysts, and sometimes several additives are necessary to achieve transition-metal-catalyzed chemical transformation. Furthermore, the elimination of trace amounts of metal catalyst from the desired product upon completion of the reaction, which is crucial in the pharmaceutical industry, is costly and frequently becomes a daunting issue. These factors associated with transition-metal-catalyzed transformations indicate the failure of an environmentally benign and sustainable synthesis.^35–37^ As a result, the development of efficient chemical procedures for the synthesis of the molecular structure providing high atom- and step-economy with the main focus to minimize or avoid the use of harmful metal catalysts, co-catalysts, and any additives is highly needed to ensure the sustainability of our environment.

In line with this, transition-metal-free catalysis has recently proven to be an extremely efficient and ecologically friendly technique for synthesizing diverse structural complexity of significant therapeutic interest. It has emerged as a promising field in synthetic organic chemistry. Organic conversions employing transition-metal-free catalyst(s) have numerous benefits: eco-friendly, simple handling in reaction, no tedious work-up procedure, toxic-free, ligand-free, metal-free, additive-free, waste-free, *etc.*^38–40^

Recognizing the significance of ultrasound irradiation as an alternative eco-friendly method and the synthetic efficiency associated with transition-metal-free catalysis, the last decades have witnessed outstanding growth in the application of sonochemical activation in the synthesis of a library of heterocyclic compounds, especially under transition-metal-free catalysis. Several review articles demonstrated the application of ultrasound irradiation in diverse synthetic organic transformations.^41–52^ Banerjee summarized the ultrasound-assisted organic reactions under catalyst-free conditions in 2017.^53^ To our delight, no review articles existed for the transition-metal-free synthesis of diverse heterocyclic compounds based on the sonochemical activation approach. Herein, we have demonstrated a current overview of the transition-metal-free synthesis of diverse five-membered and six-membered heterocycles and complex-fused and spiro-heterocycles of potential therapeutic interest under ultrasound irradiation covering the literature from 2015 to date. Besides demonstrating the remarkable progress made in this fascinating area, we have also emphasized the limitations and challenges connected with reaction discovery to encourage further research.

## 2. Ultrasound irradiation-promoted transition-metal-free synthesis of five-membered heterocycles

### 2.1 Synthesis of five-membered heterocycles containing one-heteroatom

#### 2.1.1 Synthesis of pyrroles

The well-known five-membered ring pyrroles and their analogs represent a prominent family of nitrogen-containing heterocycles frequently recognized in the architecture of marine natural products, and potential therapeutic candidates. Pyrroles, of synthetic and natural origin, are an integral structural unit in a wide variety of pharmacologically interesting compounds and they also serve as efficient building blocks for the manufacture of diverse commercially available drugs and optoelectronic devices.^54^ Considering their high chemical landscape and prolific pharmacological activity, numerous methodologies for constructing pyrroles have emerged over the last decades.

**Scheme 1:**
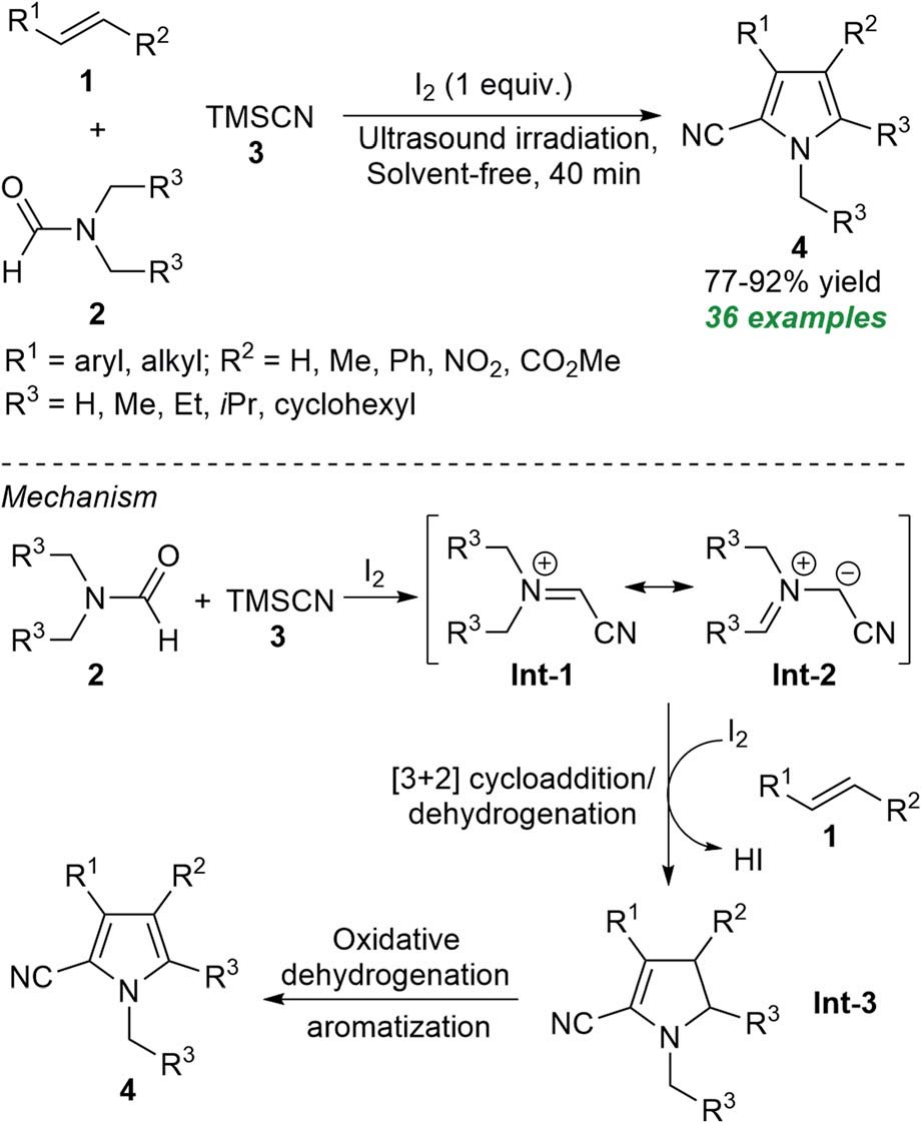
Ultrasound-assisted iodine-catalyzed one-pot three-component routes to access pyrroles 4.

From the perspective of green and more sustainable chemistry, along with the possibilities of obtaining eco-and pot economic nature of the transformation *via* the well-known multicomponent reaction approach, Gui *et al.* developed an ultrasound-assisted tandem one-pot protocol for the preparation of polysubstituted pyrroles 4 from the three-component reaction of alkenes 1, *N*,*N*-disubstituted formamides 2 and TMSCN 3 under solvent-free conditions (Scheme 1).^55^ Using iodine as the catalyst and the oxidant, the corresponding products 4 were obtained in 77–92% yield within 40 minutes. The use of ultrasound reduced the reaction time from hours to minutes, making this approach energy-efficient and environmentally friendly. The overall process can proceed through the ultrasound-assisted iodine catalyzed formation of azomethine ylide Int-1 and resonating structure Int-2 from 2 and 3 that can then undergo regioselective [3 + 2] cycloaddition with 1 followed by *in situ* dehydration to produce intermediate Int-3. The subsequent oxidative dehydrogenation and aromatization of Int-3 yield the final products 4.

**Scheme 2:**
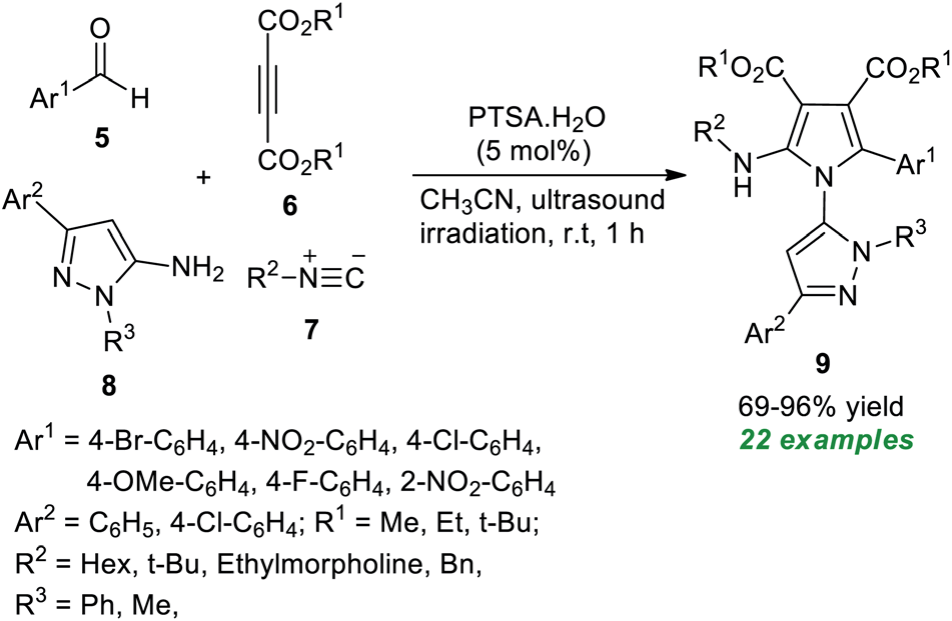
Acid-catalyzed chemoselective synthesis of pyrroles 9 under ultrasound irradiation.

In 2021, Nazeri *et al.*^56^ disclosed a highly chemoselective multicomponent strategy towards synthesizing polysubstituted pyrroles 9 by introducing ultrasound irradiation as the green energy source (Scheme 2). With the help of 5 mol% of PTSA·H_2_O as the organocatalyst, the desired products 9 derived from various aldehydes 5, dialkyl acetylene dicarboxylate 6, isocyanides 7, and 5-amino-pyrazoles 8, have been obtained in moderate to excellent yield. Among them, most of the synthesized compounds have been confirmed to have fluorescence activities. The broad substrate scope, excellent yield, operational simplicity, and short reaction time are some of the critical features of this methodology.

#### 2.1.2 Synthesis of benzofurans

Benzofurans are considered an essential set of heterocyclic compounds widely distributed in naturally occurring molecules and pharmaceuticals and possess anticancer, anti-inflammatory, antiviral, and antioxidant activities.^57^

**Scheme 3:**
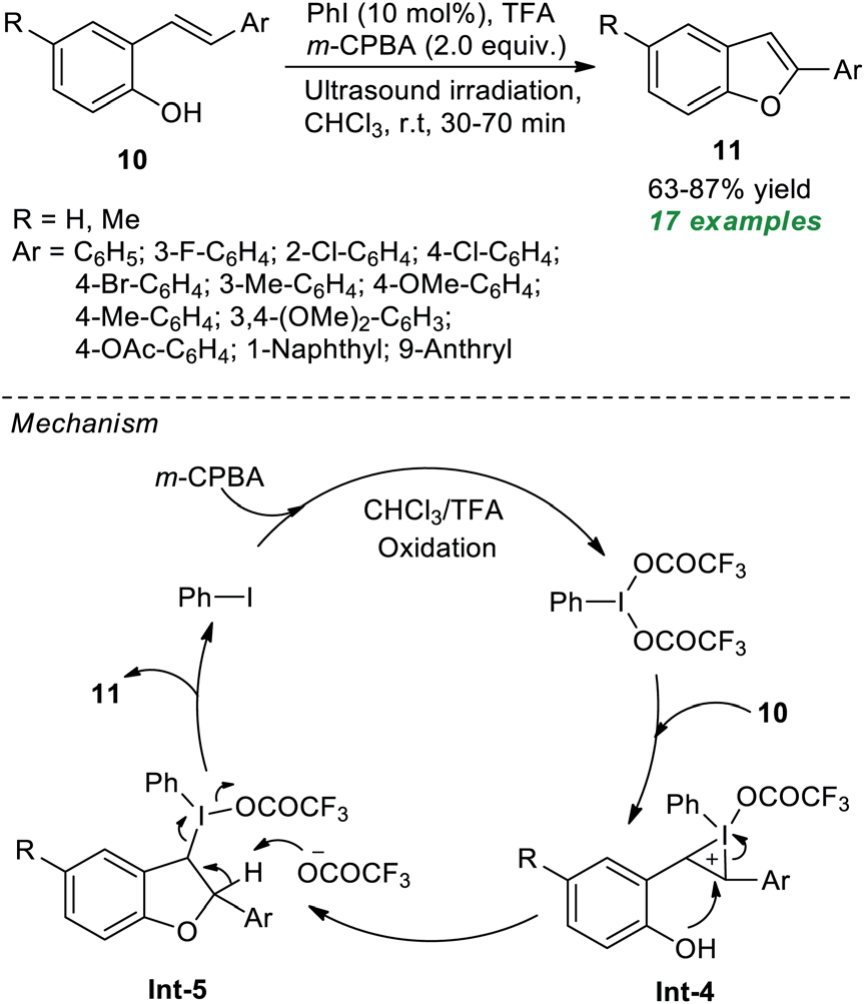
Ultrasound-assisted *in situ* generated hypervalent iodine-catalyzed synthesis of benzofurans.

In 2020, Mangaonkar and co-workers demonstrated a highly convenient ultrasound-assisted strategy for the construction of functionalized benzofurans 11*via* the cyclization of 2-hydroxystilbenes 10 (Scheme 3).^58^ Under the influence of 10 mol% of PhI as the pre-catalyst, *m*-CPBA as the oxidant, and TFA as additives, the products 11 were achieved in 63–87% yield at room temperature. In this reaction, the required iodine(iii) species were produced *in situ* from the oxidation of PhI by *m*-CPBA and TFA. This iodine(iii) species activates the C=C bond of 10 to yield intermediate Int-4, which then experiences intramolecular cyclization and subsequent reductive elimination to deliver the products 11 and precatalyst PhI *via* intermediate Int-5.

#### 2.1.3 Synthesis of thiophenes

Thiophene and its derivatives comprise a promising class of five-membered sulfur-containing heterocycles that commonly exist in oil and coal and have been known for their tremendous biological activities, including anticancer, antimicrobial, antidepressant, anti-inflammatory activity, *etc.*^59^

**Scheme 4:**
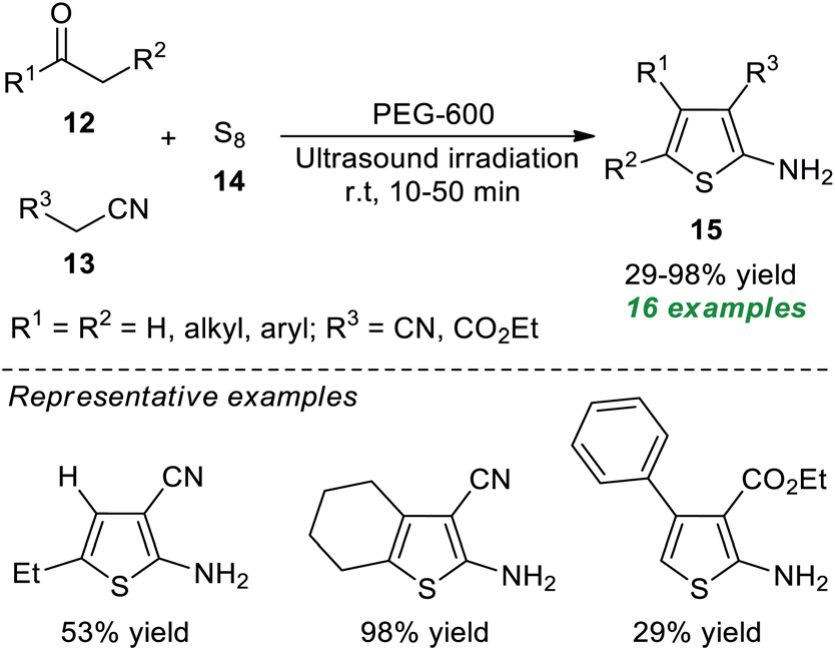
Ultrasound-assisted three-component synthesis of thiophenes 15.

Because of their importance, a rapid and eco-compatible strategy for the assembly of diverse polysubstituted thiophenes 15*via* a three-component Gewald reaction of carbonyl compounds 12, active methylene compounds 13, and elemental sulfur 14 in the presence of polyethylene glycol-600 (PEG-600) under ultrasound irradiation at room temperature was developed by Akbarzadeh and Dekamin in 2017 (Scheme 4).^60^ Using this catalyst-free protocol, 16 target compounds were accomplished in poor to excellent yield in comparatively short reaction durations.

**Scheme 5:**
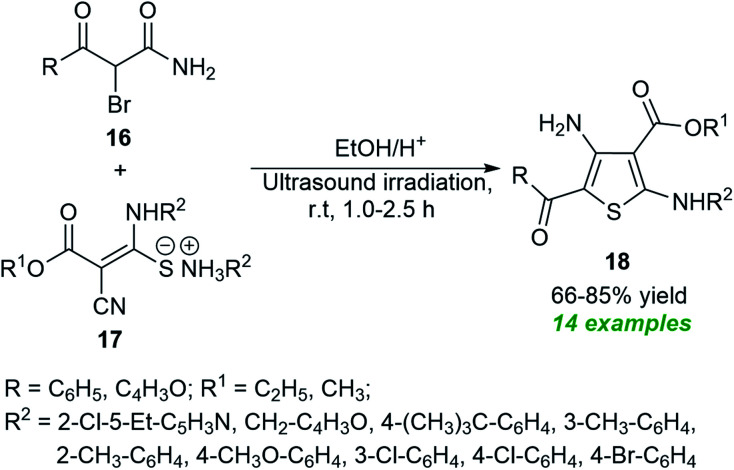
Ultrasound-assisted one-step synthesis of tetrasubstituted thiophenes 18.

In 2021, Suárez *et al.*^61^ disclosed a one-step push–pull synthetic route to access various thiophene derivatives 18 from readily available α-brominated acetamides 16 and amino mercaptoacrylates 17 by introducing ultrasound irradiation as a powerful green technique (Scheme 5). A comparison of both conventional and ultrasound irradiation techniques for the synthesis of 18 suggests the use of ultrasound condition as the method of choice, which not only increased the product yield (66–85%) but also reduced reaction times, while the conventional technique required a longer reaction time and also decreased the product yield (35–72%).

### 2.2 Synthesis of five-membered heterocycles containing two-heteroatoms

#### 2.2.1 Synthesis of substituted imidazoles

The five-membered aromatic heterocycles, imidazoles having two nitrogen atoms as well as delocalized sextet π-electrons, have received a lot of attention in many branches of chemistry as a consequence of their tremendous pharmacological applications such as anticancer, antifungal, anti-HIV, anti-inflammatory, and anti-allergic activities as well as their key role in the biological system including histidine and histamine.^62–66^

**Scheme 6:**
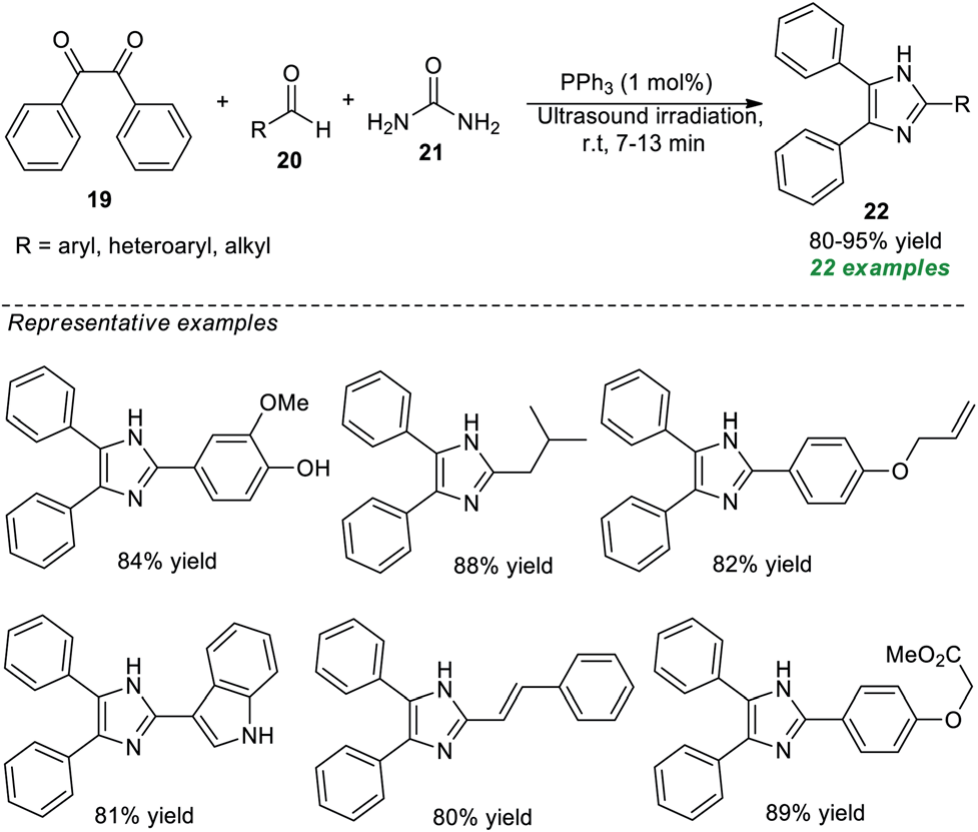
Ultrasound-assisted organocatalytic syntheses of tri-substituted imidazoles as reported by Piltan *et al.*

Recognizing their importance, various attempts to synthesize this moiety have been made. In 2020, Piltan and co-workers disclosed an organocatalytic tandem three-component approach for the facile access to trisubstituted imidazoles 22 from the treatment of benzil 19, aldehydes 20, and urea 21 in a one-pot fashion under ultrasonication (Scheme 6).^67^ A broad spectrum of substituted aromatic, heteroaromatic, and aliphatic aldehydes was well tolerated for this reaction under the influence of 1 mol% of triphenylphosphine (PPh_3_) as the organocatalyst and delivered the respective imidazole product 22 in good to high yields at room temperature. Compared to conventional heating conditions, the use of ultrasound technology enhances overall yields and reduces reaction times.

**Scheme 7:**
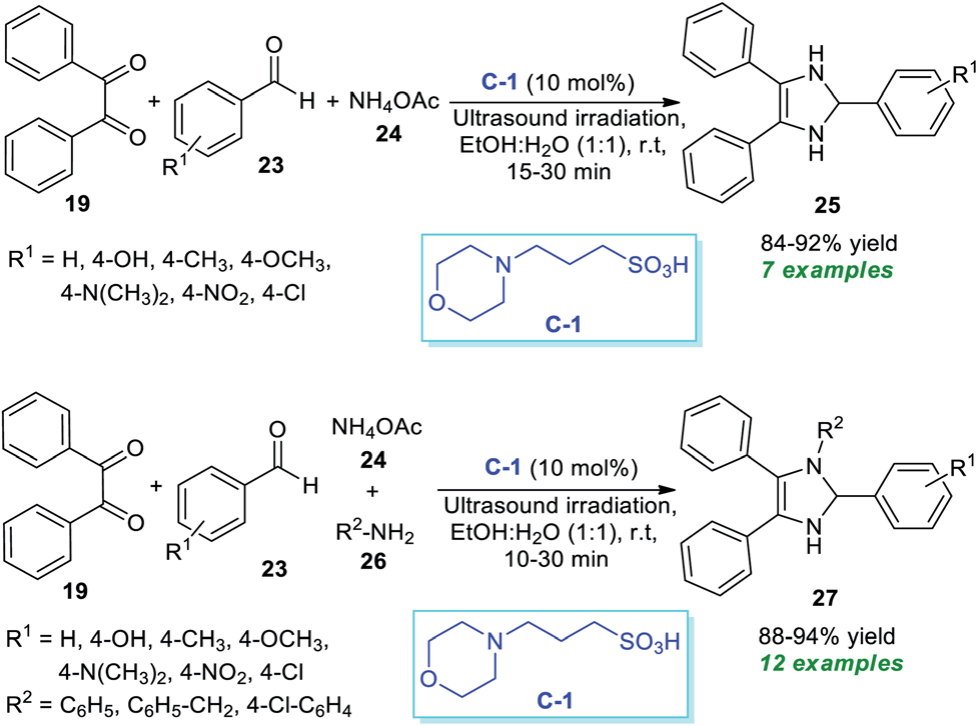
3-*N*-Morpholinopropanesulfonic acid (C-1)-catalyzed ultrasound-assisted synthesis of imidazoles.

Almost at the same time, another milestone in the synthesis of substituted imidazoles was gained by Agrawal *et al.*^68^ They disclosed a one-pot ultrasound-assisted reaction of benzil 19, aryl aldehydes 23, and ammonium acetate 24 in an aqueous ethanolic solution with 10 mol% of C-1 as the catalyst (Scheme 7). Pleasingly, this three-component reaction afforded the respective imidazoles 25 in good to high yield within 15–30 minutes. The same group further extends their three-component reaction strategy to synthesize some tetrasubstituted imidazoles. Using 10 mol% of C-1, the corresponding tetrasubstituted imidazoles 27 derived from benzil 19, aldehydes 23, ammonium acetate 24, and substituted amine 26 were formed in 88–94% yield. The exploitation of energy-saving ultrasound techniques features a clean pathway for the reaction, reduces the reaction time, and increases the product yield. Other significant aspects of this approach include the wide functional group tolerance, green reaction conditions, recyclable catalysts with low loading, *etc.* The unusually required prolonged reaction time and the low yield of the products by the thermal process make ultrasound a very attractive and powerful green strategy for this reaction in contrast to the thermal method.

#### 2.2.2 Synthesis of substituted isoxazoles

Isoxazoles and their analogs belong to a prominent class of five-membered oxygen- and nitrogen-containing heterocycles, possessing various pharmaceutical activities including analgesic,^69^ anti-cancer,^70^ COX-2 inhibitory,^71^ anti-HIV activity,^72^*etc.*

**Scheme 8:**
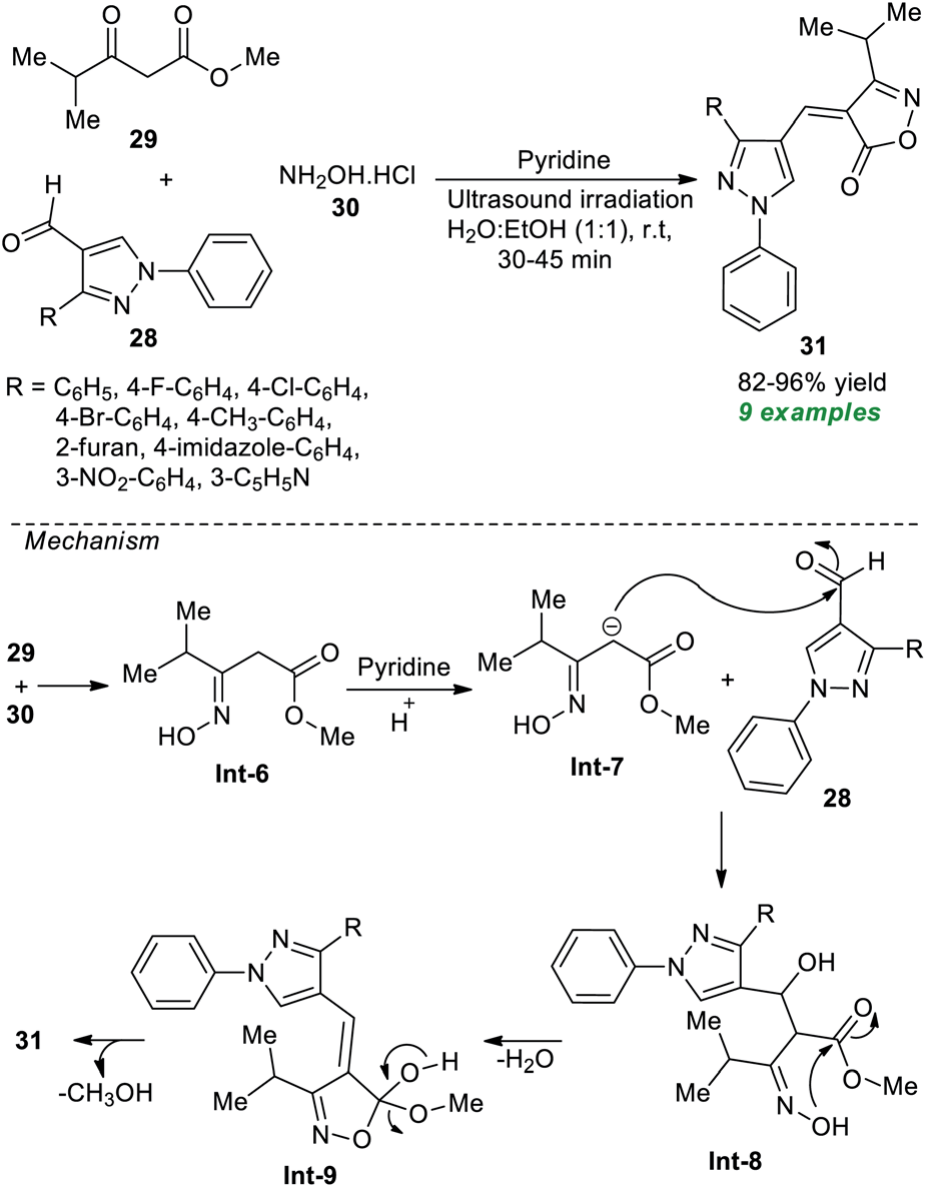
Secondary amine-catalyzed synthesis of substituted isoxazole under ultrasound irradiation.

After noticing this, many synthetic protocols have been discovered for the efficient construction of various types of isoxazoles, particularly α, β unsaturated isoxazole-5(4*H*)-one's derivatives. However, most of the existing methods deal with several significant issues due to which Joshi *et al.*^73^ in 2019 developed an ultrasound-assisted rapid, efficient one-pot three-component method for the practical synthesis of diverse isoxazole derivatives (Scheme 8). Using pyridine as the organocatalyst, the three-component reaction of several pyrazole aldehydes 28, methyl 4-methyl-3-oxovalerate 29, and hydroxylamine hydrochloride 30 in aqueous ethanolic solution at room temperature yielded a total of 9 new novel isoxazole derivatives 31 with 82–96% yields. A comparison study between the conventional and ultrasound conditions concluded the ultrasound method as the best efficient method in terms of product yield and reaction times. The mechanism begins with the formation of intermediate Int-6 from the reaction of 29 and 30, which undergo nucleophilic addition with 28 under the influence of pyridine to deliver the intermediate Int-8. The final products 31 have been achieved *via* cyclization of Int-8 followed by elimination of water and methanol.

**Scheme 9:**
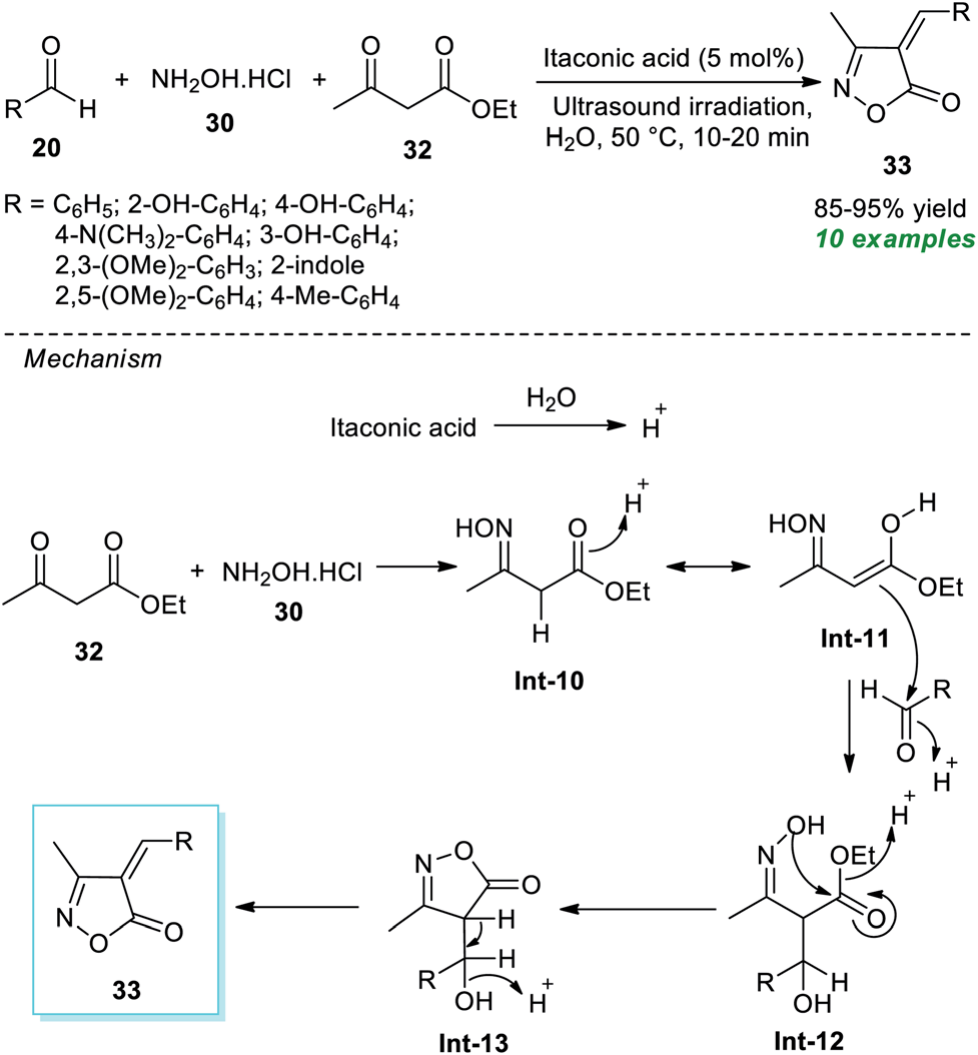
Itaconic acid-catalyzed three-component synthesis of isoxazoles under ultrasound irradiation.

Around the same time, Thopate and Kasar also demonstrated an expedient organocatalytic one-pot technique to produce various isoxazole-5(4*H*)-one derivatives (Scheme 9).^74^ The three-component reaction of a variety of aldehydes 20, hydroxylamine hydrochloride 30, and ethyl acetoacetate 32 in water as the solvent at 50 °C using 5 mol% of itaconic acid as the organocatalyst was found to proceed smoothly to form the corresponding isoxazole derivatives 33 in 85–95% yields in a concise duration of time under ultrasonication. Broad functional group tolerance and a reusable catalytic system with low loading and metal- and waste-free nature are several key highlights of this approach. The author postulated a mechanism to realize this transformation, which entails the generation of the key intermediate Int-11*via* acid-mediated reactions of 30 and 32. This intermediate Int-11 reacts with 20 to produce Int-12, which further experiences intramolecular cyclization under the influence of itaconic acid to yield the final products 33.

#### 2.2.3 Synthesis of substituted oxazoles

The five-membered oxazole ring, frequently encountered in numerous natural products and bioactive heterocyclic scaffolds, has attracted much more interest in the domain of medicinal chemistry owing to its prolific bioactivity profile.^75^

**Scheme 10:**
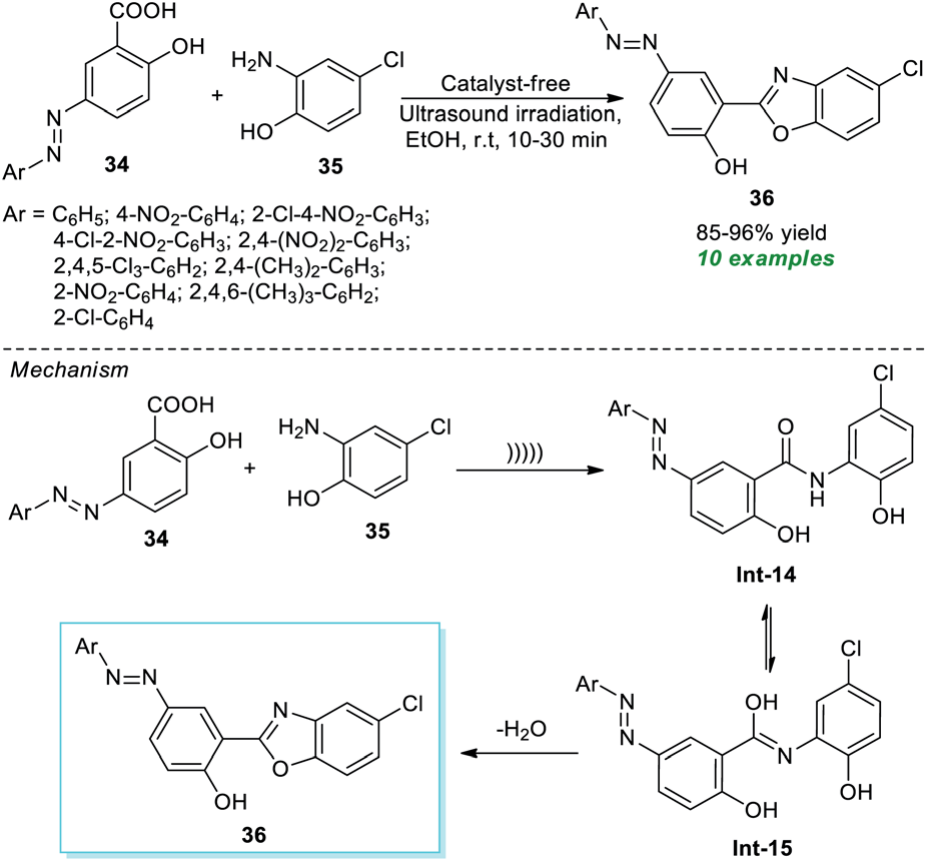
Ultrasound-assisted azo-linked benzoxazoles as reported by Nikpassand *et al.*

In this regard, Nikpassand *et al.*^76^ developed a facile one-pot procedure for the construction of a series of novel benzoxazole derivatives with the help of ultrasound irradiation (Scheme 10). Using the catalyst-free conditions, the reaction of various azo-linked salicylic acid derivatives 34 and 2-amino-4-chlorophenol 35 in ethanol was found to proceed under ambient conditions under ultrasound irradiation to deliver highly functionalized benzoxazole derivatives 36 in 85–96% yields in 10–30 minutes. The primary advantages of this approach include cost-effectiveness, operational simplicity, sustainability, *etc.* A mechanism is depicted in Scheme 10 for this reaction. Initially, an intermediate Int-14 is formed from the nucleophilic addition of 35 to 34 under ultrasonication. Consequently, the tautomerization of Int-14 to Int-15 and its dehydration lead to the desired products 36.

**Scheme 11:**
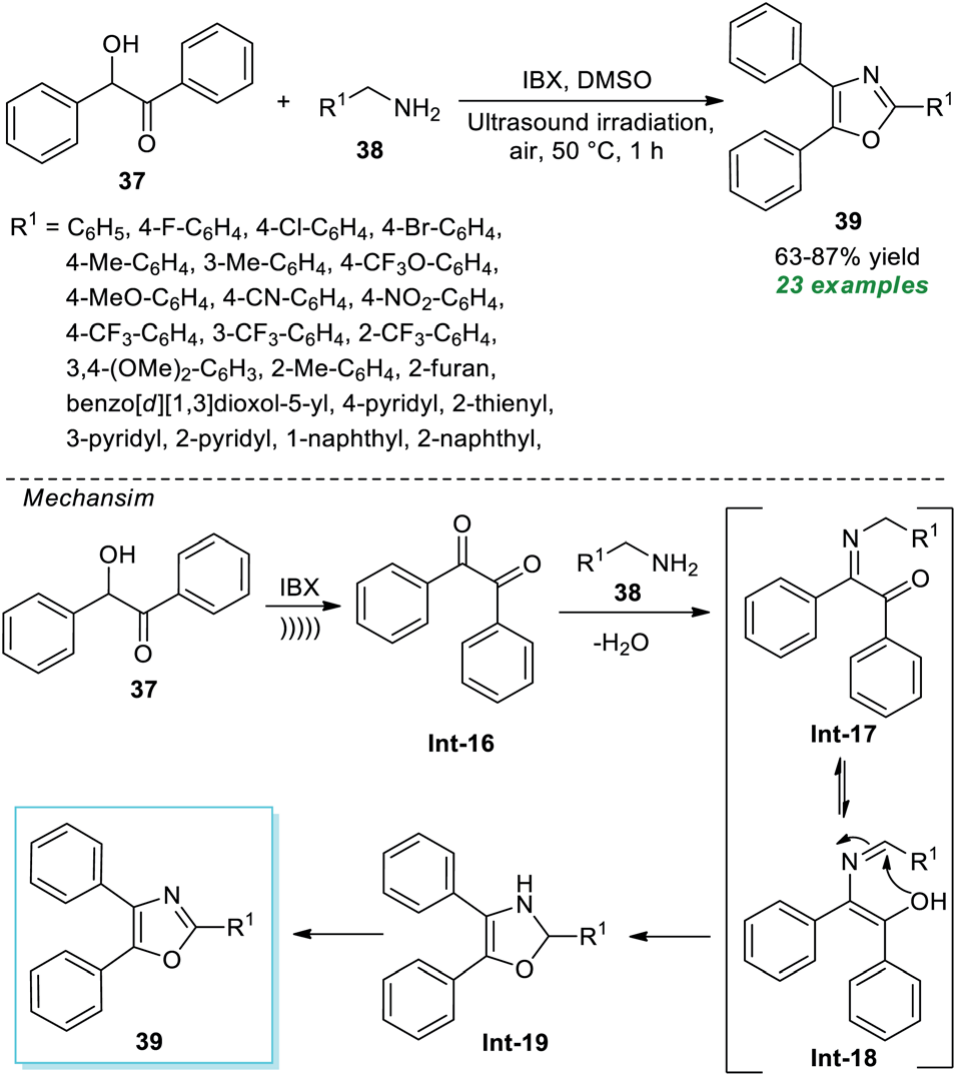
IBX-mediated synthesis of 2-aryl substituted oxazoles under ultrasound irradiation.

Another achievement in the synthesis of oxazoles derivatives was accomplished by Dandela, Pal, and their group in 2021 by employing the ultrasound technique as an efficient green strategy. Treatment of commercially available benzoin 37 with various amines 38 in the presence of IBX was found to occur in DMSO under air at 50 °C to deliver the respective oxazole derivatives 39 in 63–87% yields (Scheme 11).^77^ This method was successfully proceeded not just with aryl amines but also with alkyl and heteroaryl amines. The overall reaction could be initiated *via* the IBX mediated transformation of benzoin 37 to benzil Int-16 and then reaction with amines 38 to afford the adduct Int-17 which after tautomerization and intramolecular cyclization delivers Int-19. Its final aromatization in the presence of air yields the desired products 39.

#### 2.2.4 Synthesis of substituted pyrazoles

In recent decades, pyrazoles and pyrazolones, the five-membered nitrogen-containing compounds, have received immense interest among synthetic chemists because of their broad-spectrum application in synthetic organic chemistry, medicinal chemistry, materials science, food industry, cosmetics, and so on.^78,79^

**Scheme 12:**
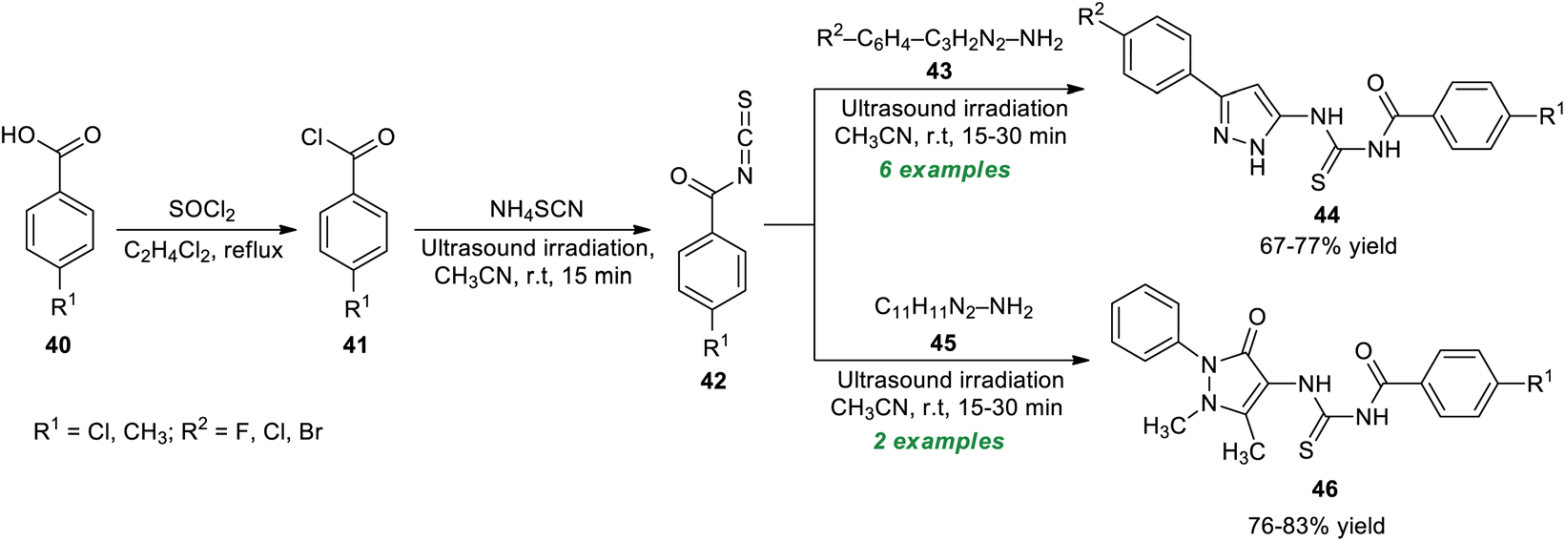
Ultrasound-assisted step-wise synthesis of thiourea-linked pyrazole derivatives.

Considering these importances, Bleotu *et al.*^80^ demonstrated an eco- and environmentally benign strategy for the construction of diverse pyrazoles and pyrazolone derivatives under ultrasound irradiation conditions (Scheme 12). The overall synthetic process involves the initial reaction of substituted benzoic acid 40 with thionyl chloride, which delivers the desired benzoyl chloride 41. The ultrasound-assisted reaction of 41 with ammonium isothiocyanate in CH_3_CN at room temperature provided benzoyl isothiocyanates 42. The definitive treatment of 42 with 43 or 45 yields the corresponding pyrazole products 44 and pyrazolone products 46 in 67–77% and 76–83% yield, respectively. Many synthesized compounds have been recognized as the inhibitors of cell cycle kinases that mark the key features of the present protocol.

**Scheme 13:**
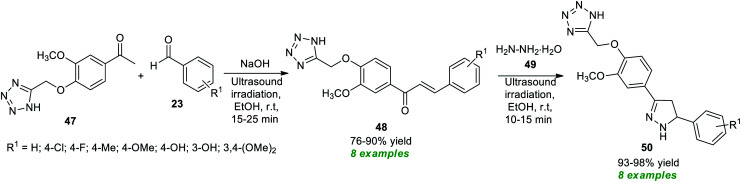
One-pot synthesis of tetrazole-linked pyrazole derivatives under ultrasound irradiation.

Recently, Sarkate and co-workers have devised the synthesis of a library of tetrazole-based pyrazoline derivatives by employing ultrasound irradiation as an eco-friendly activation method with the aid of metal-free conditions, and the synthesized compounds were found to exhibit anticancer properties (Scheme 13).^81^ The desired pyrazoline products were achieved *via* a two-step procedure starting from the initial Claisen–Schmidt condensation of tetrazole linked carbonyl compound 47 and various aldehydes 23 in ethanol using NaOH as the base catalyst at room temperature to afford the adduct 48 in 76–90% yields in a short duration of time, which on further treatment with hydrazine hydrate 49 under ultrasound irradiation offers the respective products 50 in 93–98% yields.

#### 2.2.5 Synthesis of substituted thiazoles

**Scheme 14:**
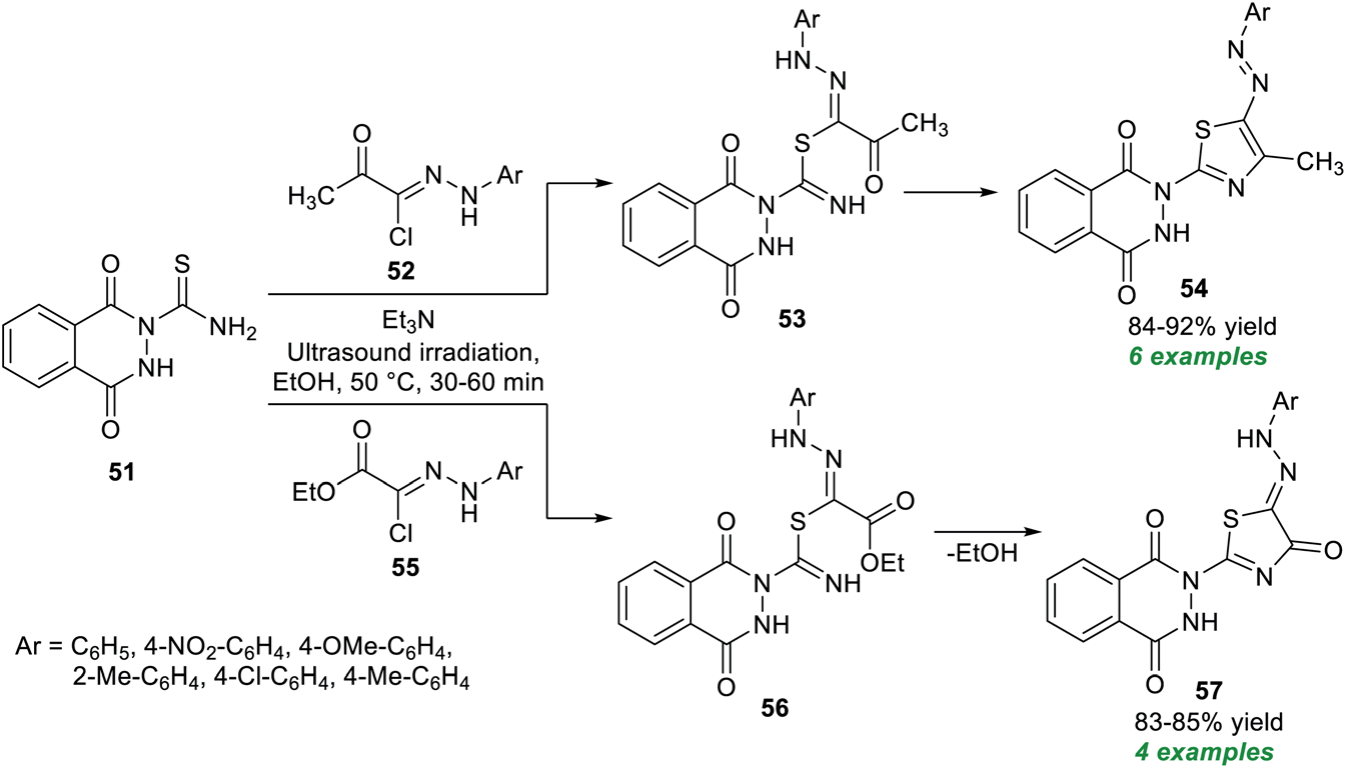
Ultrasound-assisted triethylamine-catalyzed assembly of functionalized thiazoles as reported by Farghaly *et al.*

The thiazole skeleton is considered one of the essential five-membered heterocycles due to its common prevalence in the basic framework of naturally existing compounds and bioactive molecules including vitamin B_1_, penicillin, bleomycin, *etc.*^82^ To realize this significance, a very beneficial and straightforward method for expedient access to various thiazole derivatives was developed by Farghaly *et al.* (Scheme 14).^83^ By using a tertiary amine Et_3_N as the catalyst, the corresponding thiazole derivatives 54 and 57 derived from the ultrasound-assisted reaction of thioamide 51 and acetyl hydrazonoyl chloride 52 or ethyl (*N*-arylhydrazono)-chloroacetates 55 have been accomplished in 84–92% and 83–85% yield respectively at 30–60 minutes. Compared to the traditional thermal method, ultrasound techniques allow for easy and clean isolation of the products in pure form in a relatively quick reaction time. On the other hand, the limited substrate scope indicates a shortcoming of the present protocol otherwise incredible development.

**Scheme 15:**
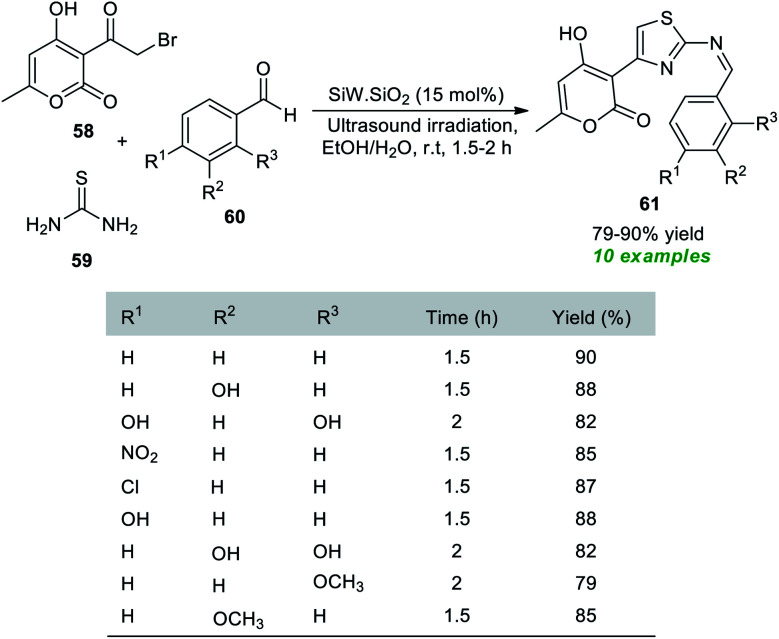
SiW·SiO_2_-catalyzed ultrasound-irradiated assembly of thiazole derivatives.

A simple, facile, and highly convenient method to access a variety of Hantzsch thiazole derivatives has been reported by the Rachedi group.^84^ With the help of their prepared silica-supported tungstosilisic acid (SiW·SiO_2_) catalyst, a one-pot three-component reaction between bromoacetyl substituted-pyran-2-one 58, thiourea 59, and aryl aldehydes 60 was performed with ultrasound irradiation in aqueous ethanolic solution, which delivers the desired thiazole derivatives 61 in 79–90% yield at room temperature within 1.5–2 hours (Scheme 15). Substitutions in different positions of the benzaldehyde rings revealed no significant influence on the reaction rate or product yield.

### 2.3 Synthesis of five-membered heterocycles containing three-heteroatoms

#### 2.3.1 Synthesis of oxadiazoles

**Scheme 16:**
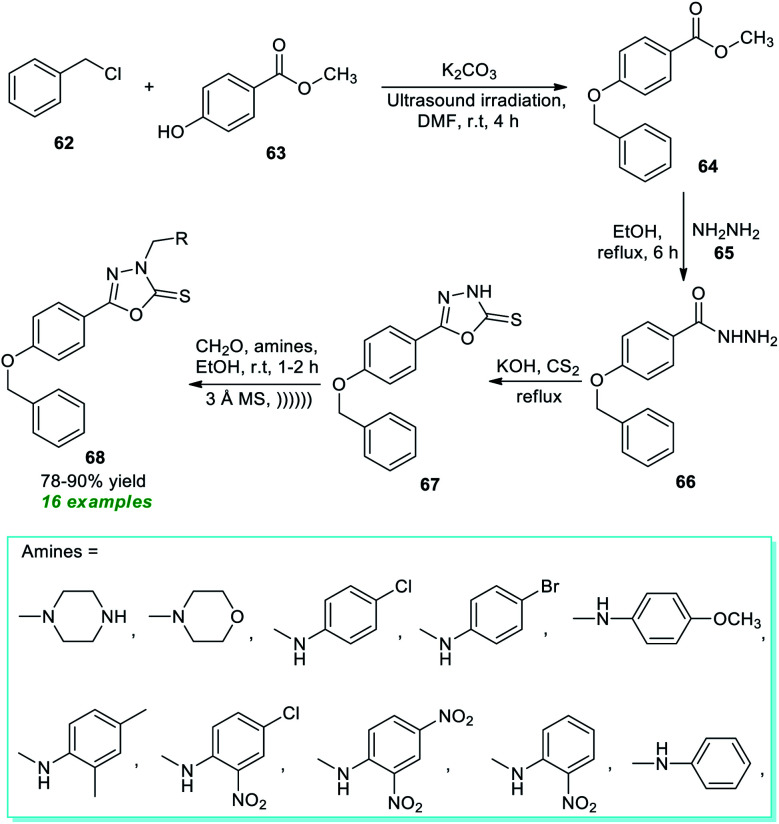
Step-wise synthesis of diverse 1,3,4-oxadiazoles under ultrasound irradiation.

Owing to the broad synthetic landscape and immense pharmacological activities, the five-membered three heteroatom (including one oxygen and two nitrogen atoms) containing heterocycle, oxadiazole, and their derivatives have been recognized as a key motif in the discovery of new drugs. They have drawn a lot of interest in medicinal and organic chemistry over the past decades.^85^ As a consequence, Nikalje *et al.*^86^ developed an ultrasound- and molecular sieve-assisted step-wise method for the construction of diverse highly functionalized 1,2,3-oxadiazole derivatives (Scheme 16). Using K_2_CO_3_ as the base catalyst, the initial reaction of benzyl chloride 62 and methyl 4-hydroxybenzoate 63 was found to proceed under ultrasonication in DMF at room temperature to form methyl 4-(benzyloxy)benzoate 64, which was then refluxed with hydrazine hydrate 65 in the next step, leading to 4-(benzyloxy)benzohydrazide 66 as the single product. Subsequent reaction of 66 with carbon disulfide using KOH as the base under reflux conditions afforded *N*-unsubstituted oxadiazoles 67, which on treatment with various amines and formaldehyde in the presence of activated molecular sieves by employing ultrasonication delivers the final substituted 1,3,4-oxadiazole products 68 in moderate to good yield.

**Scheme 17:**
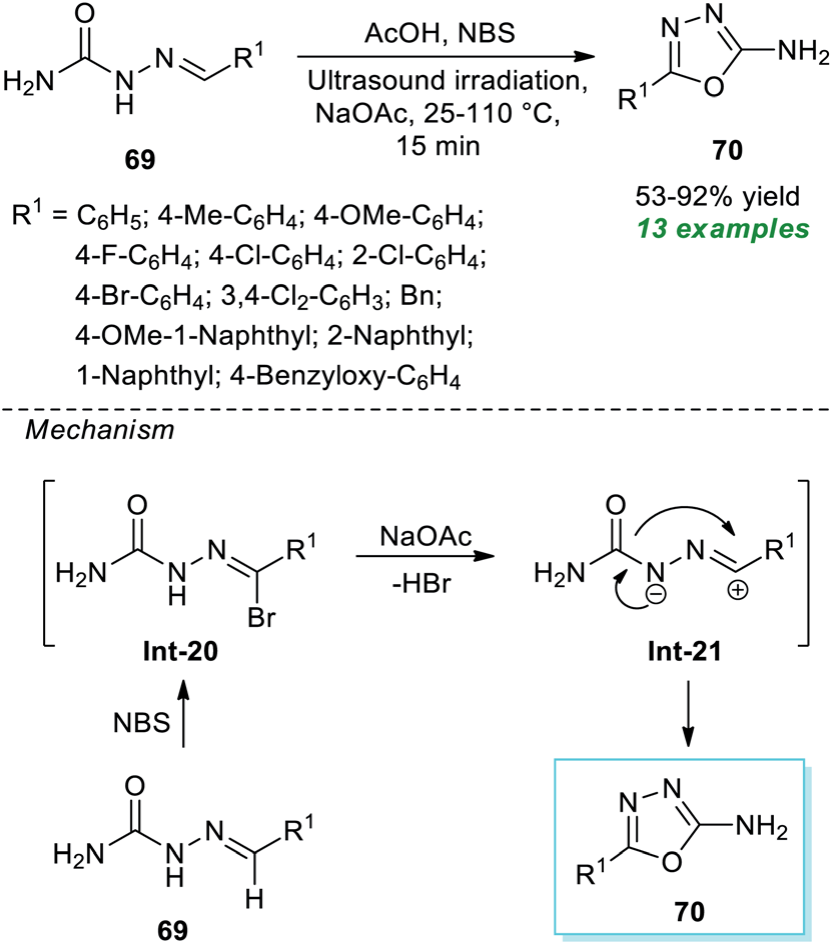
Ultrasound-assisted oxidative cyclization approach to access amino-substituted oxadiazoles.

A very straightforward method to access a variety of 1,3,4-oxadiazole derivatives under ultrasound irradiation was reported by Santos, Machado, and their group (Scheme 17).^87^ Using NBS-NaOAc as the oxidizing system, the corresponding 1,3,4-oxadiazole products 70 derived from the cyclization of various semicarbazones 69 in the presence of acetic acid at 25–110 °C were obtained in 53–92% yield within only 15 minutes. This protocol starts with the initial NBS promoted reaction of semicarbazones 69 to form an intermediate Int-20 which, after 1,3-dipolar elimination on treatment with NaOAc, delivers nitrilimines Int-21. Consequently, the 1,5-electrocyclization of the adduct Int-21 furnishes the desired products 70. Broad functional group tolerance, simple work-up procedure, eco-friendly nature, scalable synthesis, and being environmentally benign are a few glimpses of the protocol's most essential features.

**Scheme 18:**
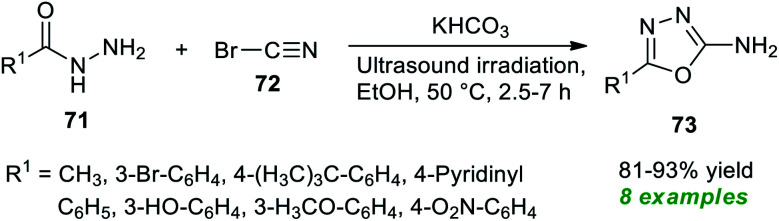
Potassium bicarbonate-catalyzed ultrasound-assisted synthesis of 1,3,4-oxadiazole derivatives.

Treatment of various hydrazides 71 and cyanogen bromide 72 in the presence of potassium bicarbonate as an efficient catalyst in ethanol under ultrasonication at 50 °C was found to lead to a variety of 1,3,4-oxadiazole derivatives 73 in 81–93% yields after 2.5–7 hours (Scheme 18).^88^ Alkyl and heteroaryl substituted aldehydes situated on the hydrazide ring have been well sustained by this approach, like aromatic aldehydes with varied electron-withdrawing and electron-donating groups. The significant outcome of the present method was established by authenticating the antioxidant properties of most of the synthesized compounds.

#### 2.3.2 Synthesis of thiadiazoles

**Scheme 19:**
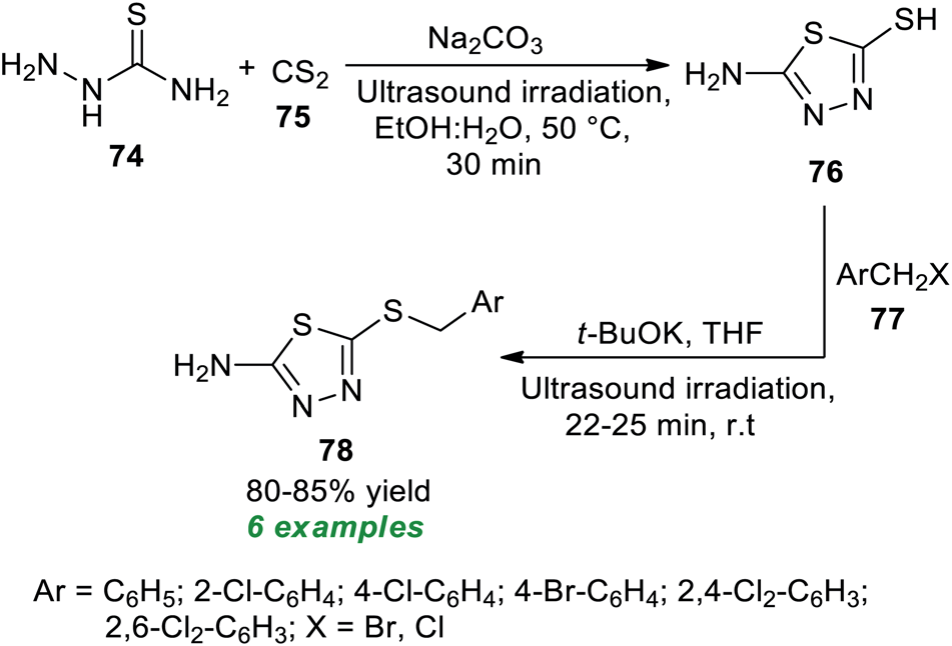
Ultrasound-assisted synthesis of amino-substituted thiadiazole scaffolds.

Thiadiazoles and their derivatives hold great potential in pharmaceutical chemistry as a consequence of their broad-spectrum therapeutic efficacies, including antimicrobial, antituberculosis, anti-inflammatory, and anticancer. Furthermore, they have wide applications in optics and electrochemistry.^89^ To realize this importance, Erdogan in 2019 developed an efficient, rapid, and facile strategy for the construction of various amino-substituted thiadiazole products 78 with the aid of metal-free catalysis under ultrasound irradiation (Scheme 19).^90^ The overall synthetic procedure starts with the initial ultrasound irradiated reaction of thiosemicarbazide 74 and carbon disulfide 75 in an aqueous ethanolic solution under the influence of a base catalyst sodium carbonate at 50 °C for 30 minutes to afford thiadiazole-2-thiol 76, which on treatment with different aryl halides 77 in the presence of potassium-*tert*-butylate in THF under sonication at room temperature delivers the corresponding amino-substituted thiadiazole derivatives 78 in good yields. The short reaction time, energy efficiency, ease of set-up, low cost, and lack of waste support the sustainability and eco-friendly nature of the protocol.

**Scheme 20:**
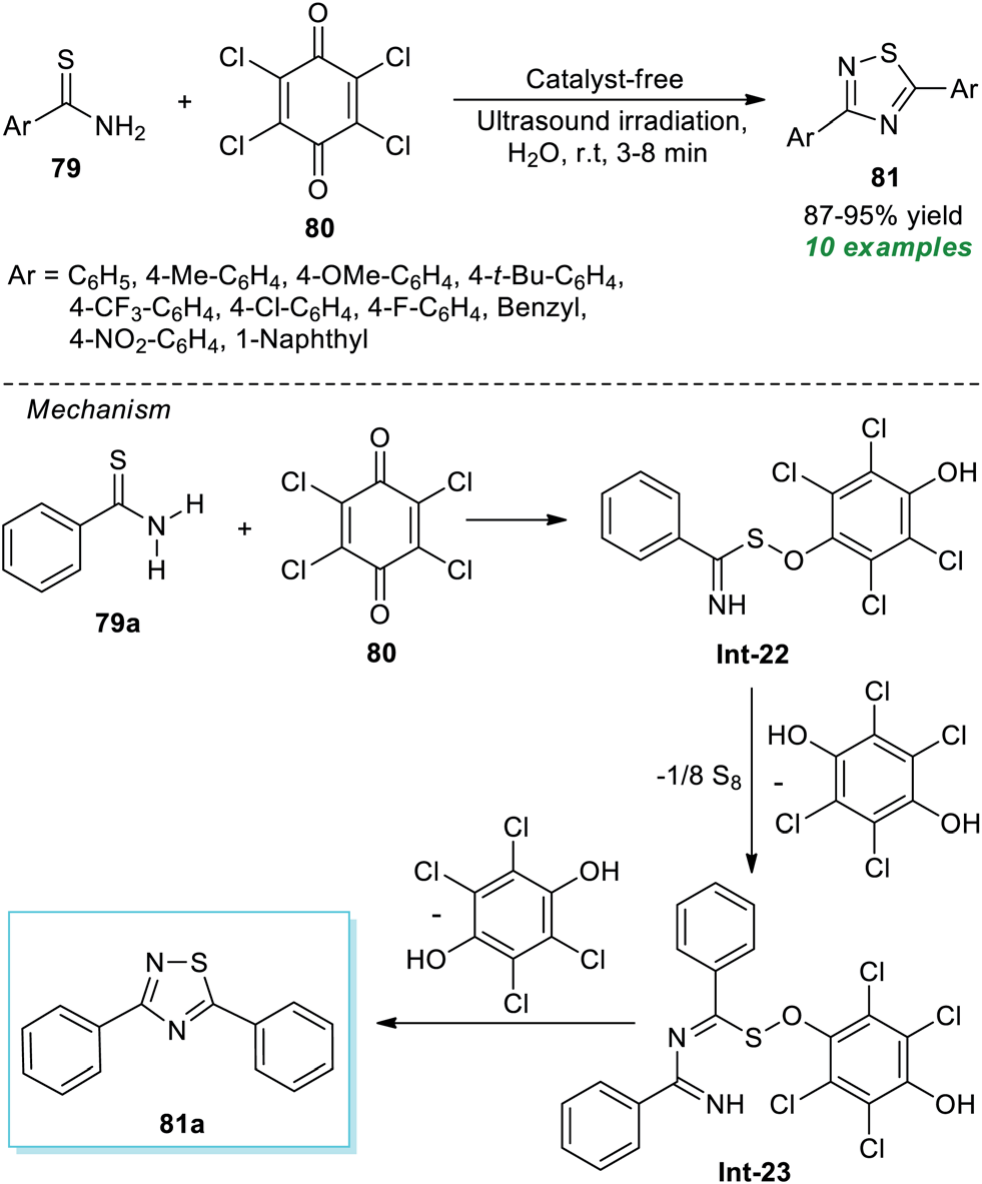
Ultrasound-assisted conversion of thioamides to thiadiazoles in an aqueous medium.

Recently, a simple and expeditious method for the green synthesis of several 1,2,4-thiadiazole derivatives has been accomplished by Srivastava *et al.*^91^ by employing metal-as well as catalyst-free and ultrasound irradiation as the eco-friendly and environmentally benign reaction condition (Scheme 20). With the help of their optimized reaction condition, the corresponding thiadiazole derivatives 81 derived from thioamides 79 and chloranil 80 have been obtained in good to excellent yield in a water medium. By employing ultrasound techniques, a total of ten products were isolated in a short interval of time. The proposed mechanism for this reaction involves the oxidative addition of thioamide 79a to chloranil 80 to form an intermediate Int-22 which dimerizes to generate the intermediate Int-23. Consequently, the cyclization of intermediate Int-23 yields the final products 81.

#### 2.3.3 Synthesis of triazoles

Triazoles and their derivatives, which are nitrogen-based five-membered heterocycles, are abundantly dispersed in the architecture of naturally occurring and synthetic bioactive components.^92^ Considering their significance and chemists' ever-increasing imagination of innovative transformations of this product, a diverse set of synthetic procedures were developed in the previous decades.^93^

**Scheme 21:**
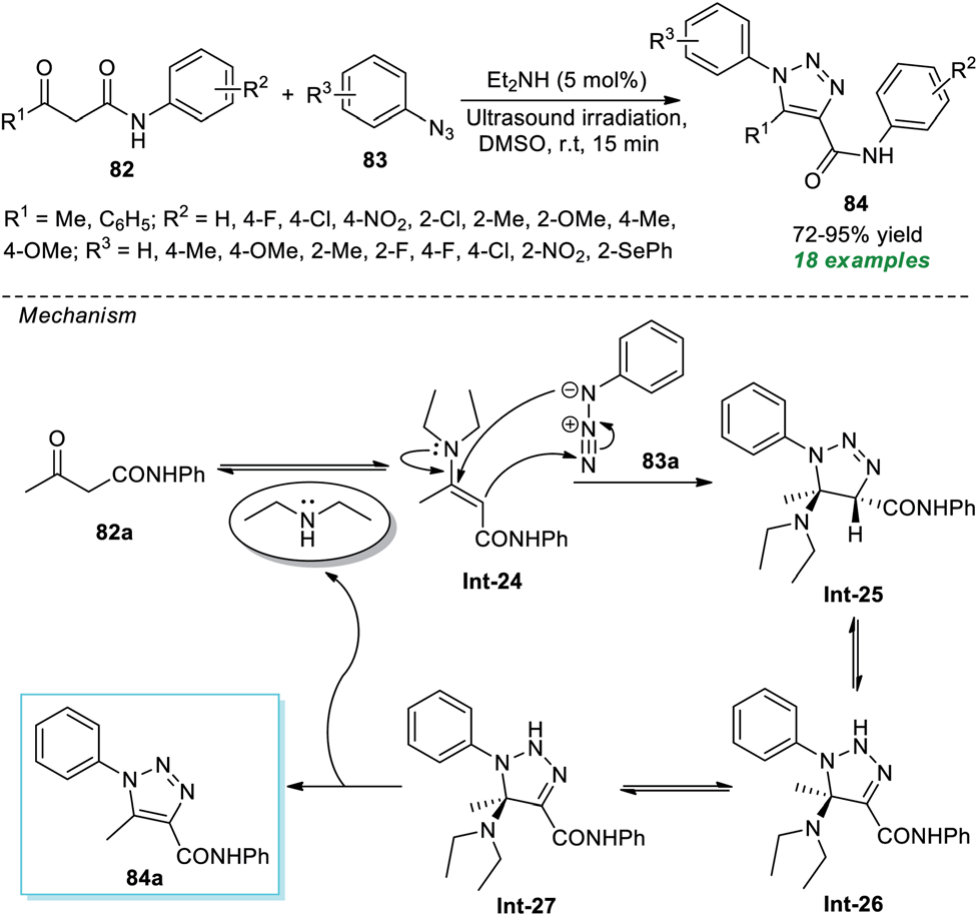
Organocatalytic ultrasound-assisted synthesis of substituted 1,2,3-triazole derivatives.

In 2017, Alves *et al.*^94^ disclosed an organocatalytic [3 + 2] cycloaddition reaction for the rapid access to a variety of 1,2,3-triazole derivatives under ultrasound irradiation (Scheme 21). The corresponding triazole products 84 produced from a variety of β-oxo-amides 82 and substituted aryl azides 83 were achieved in reasonable to outstanding yields by employing 5 mol% of diethylamine as an organocatalyst. The appearance of an electron-withdrawing group on the aryl ring of β-oxo-amides reduces the product yield, while an electron-releasing substituent enhances it. On the other hand, aryl azides bearing different substituents seemed to have no negative impact on the rate of the reaction except methyl- and fluoro-substituted aryl azide (R^3^ = 4-Me, 2-F), which offers the product with a lower yield. The mechanistic route for realizing this transformation begins with the generation of enamine intermediate Int-24 from the condensation of Et_2_NH and 82a, which then undergoes 1,3-dipolar cycloaddition with 83a to yield intermediate Int-25. Following the removal of Et_2_NH, Int-25 experiences a 1,3-hydride shift to generate the intermediate Int-26, which rapidly undergoes its zwitterionic form Int-27 and produces the desired product 84a.

**Scheme 22:**
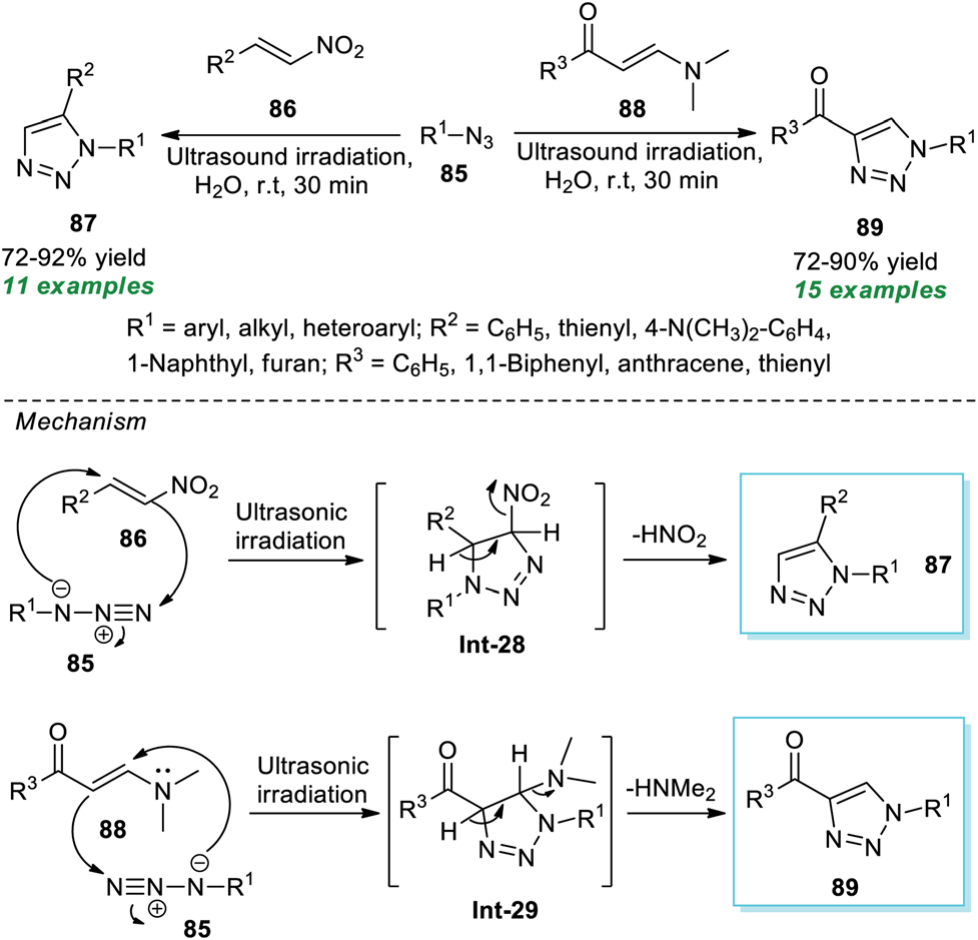
Water-mediated sonochemical-assisted synthesis of 1,5- and 1,4-substituted 1,2,3-triazoles.

Recently, Karthikeyan *et al.*^95^ demonstrated a convenient and straightforward strategy to access a vast array of 1,5-substituted triazoles 87 and 1,4-substituted triazoles 89 (Scheme 22). By employing metal- and catalyst-free conditions, the ultrasound-assisted treatment of various azides 85 and nitroolefins 86 or β-enaminones 88 with water as the green solvent at room temperature efficiently produced the respective products 87 and 89 in 72–92% and 72–90% yield respectively at 30 minutes. A range of alkyl, aryl, and heteroaryl substituted azides were discovered to operate very well with this approach. The formation of 87 was proposed *via* the key intermediate Int-28 generated by a regioselective [3 + 2] cycloaddition of azide 85 with nitroolefins 86 and the subsequent aromatization of Int-28. In the case of products 89, an inverse-electron-demand [3 + 2] cycloaddition between azide 85 and β-enaminones 88 was proposed to occur to form the stable key intermediate Int-29, which leads to the production of the preferred product 89 once HNMe_2_ was removed.

### 2.4 Synthesis of five-membered heterocycles containing four-heteroatoms

#### 2.4.1 Synthesis of tetrazoles

As a nitrogen-containing heterocycle, tetrazoles have a significant contribution to the drug discovery and development field. They are widely used in synthesizing commercially available drugs pranlukast, pemirolast, losartan, and candesartan.^96^ Furthermore, they are widely applied in high-density energy materials and explosives.^97,98^

**Scheme 23:**
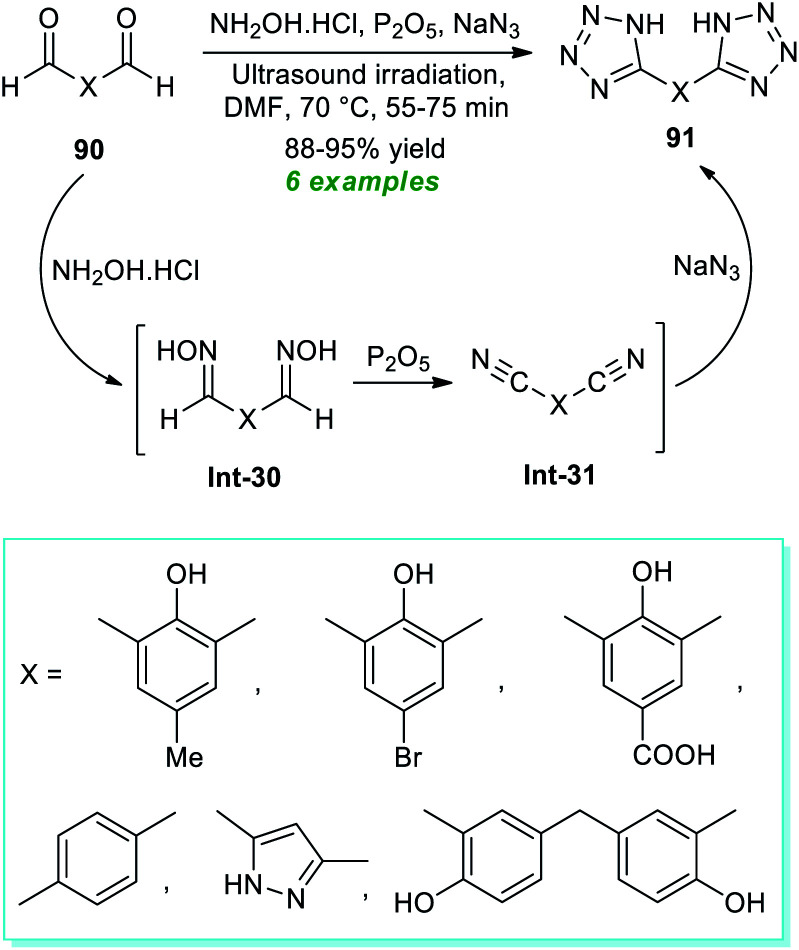
Ultrasound-irradiated one-pot synthesis of bis-tetrazole derivatives.

In 2017, Arafa *et al.*^99^ demonstrated a one-pot procedure for synthesizing bis-tetrazoles 91 from the ultrasound irradiated sequential reaction of dialdehydes 90, hydroxylamine hydrochloride, phosphorous pentoxide, and sodium azide exploring transition-metal-free conditions in dry DMF at 70 °C for 55–75 minutes (Scheme 23). By employing this mild reaction condition, six compounds have been accomplished in 88–95% yields without using column chromatography techniques. The entire reaction can begin with the generation of oximes Int-30 from dialdehydes 90 and hydroxylamine, which can react *in situ* with phosphorous pentoxide to form bis-nitriles Int-31. The final reaction of sodium azide with bis-nitriles Int-31 afforded the corresponding bis-tetrazoles 91.

**Scheme 24:**
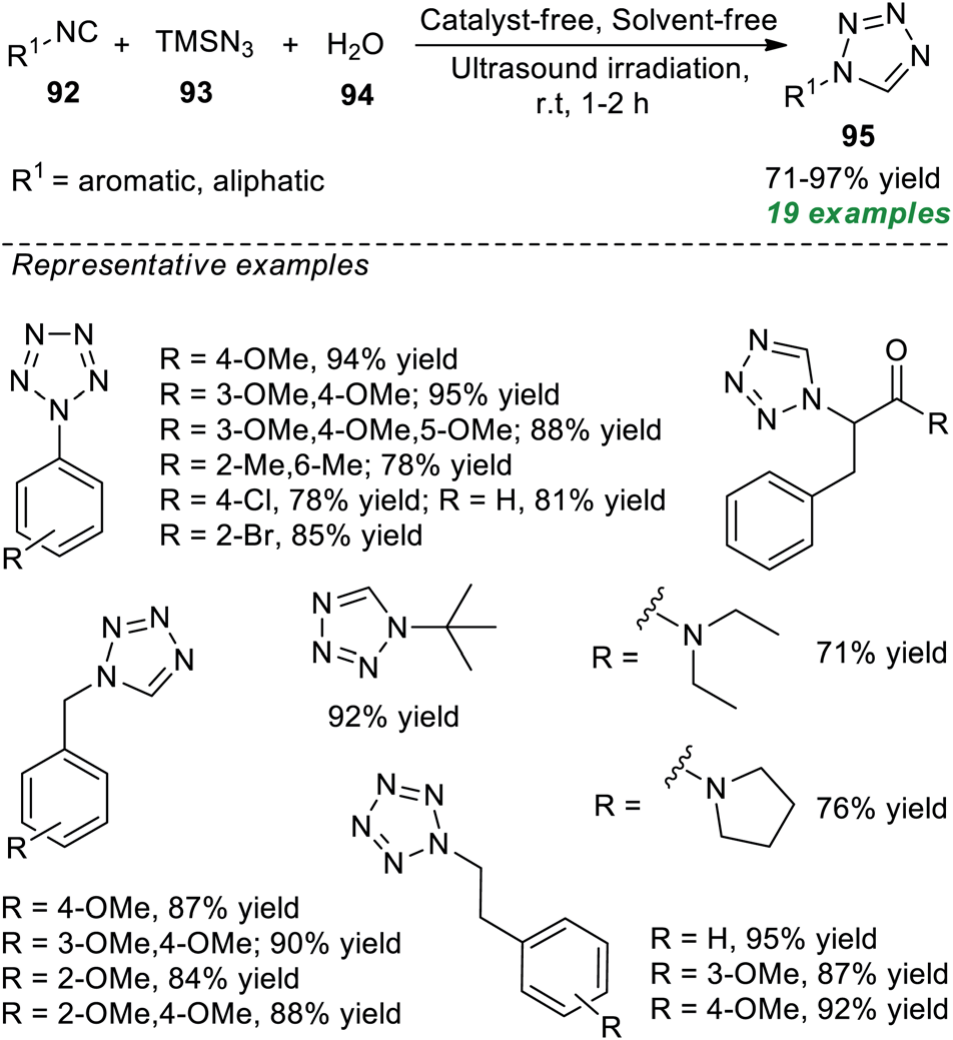
Tetrazoles obtained by the ultrasound-assisted isocyanide-based multicomponent click reaction.

Later, an isocyanide-based multicomponent click reaction towards constructing diverse substituted tetrazole derivatives by employing ultrasound techniques under catalyst-and solvent-free conditions was devised by Gámez-Montaño and co-authors (Scheme 24).^100^ With the help of their optimized reaction conditions, the corresponding tetrazole products 95 were derived from various aromatic and aliphatic substituted isocyanides 92, TMSN_3_93, and water 94; the products were unaffected by the presence of different substituents on various positions of the isocyanide ring. Broad functional group tolerance, reduced reaction time, simple handling, mild set-up, and column-free are some salient features of this strategy.

## 3. Ultrasound irradiation-promoted transition-metal-free synthesis of six-membered heterocycles

### 3.1 Synthesis of six-membered heterocycles containing one-heteroatom

#### 3.1.1 Synthesis of substituted pyridines

Highly substituted nitrogen-containing six-membered heterocycle pyridines and their fused analogs are considered privileged scaffolds, being present in many natural products and therapeutic candidates.^101–107^ The presence of this scaffold constitutes numerous biological activities including antiviral, antimicrobial, fungicidal, an inhibitor of HIV-1 integrase, *E. coli* DNA gyrase, A_2A_ adenosine receptor antagonists, *etc.*^108–113^ Consequently, the construction of highly substituted pyridines based on green or sustainable chemistry has emerged in recent years.^114^

**Scheme 25:**
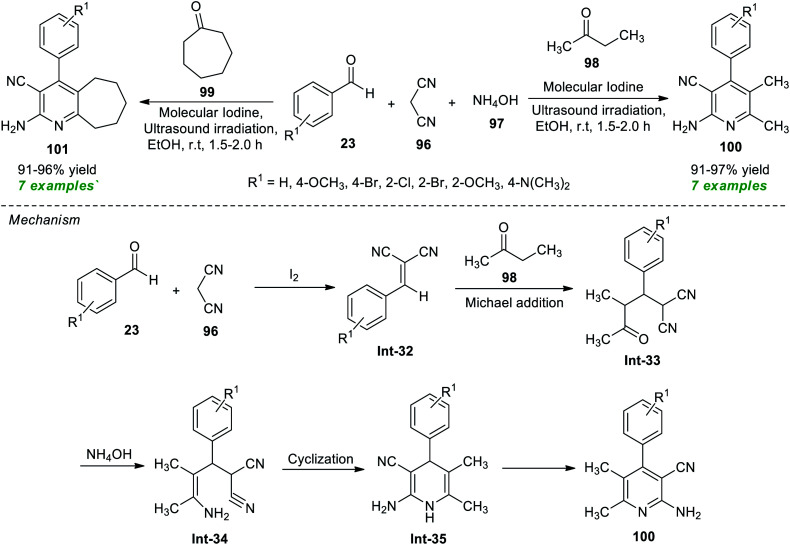
Iodine-catalyzed ultrasound-assisted multicomponent synthesis of substituted pyridines.

In a continuous effort to establish an eco-friendly and environmentally benign protocol, Pagadala *et al.*^115^ in 2020 disclosed a one-pot ultrasound-assisted multicomponent strategy for the construction of various diversely substituted pyridine derivatives (Scheme 25). The authors initially performed a four-component reaction between benzaldehyde 23, malononitrile 96, ammonium hydroxide 97, and ethyl methyl ketone 98 or cycloheptanone 99 in the presence of different catalyst systems such as acetic acid, gold, MgO, and iodine, and other solvent systems such as ethanol and acetonitrile under sonication at room temperature. The observation discovered that the reaction comprising iodine as a catalyst and ethanol or acetonitrile as a solvent efficiently offered high yields of the requisite compounds. Although both solvent systems worked well in the present protocol, ethanol has been recognized as the ideal medium to realize this reaction based on a green chemistry point of view, as acetonitrile was not recommended as a green solvent. With these conditions in hand, various substituted aryl aldehydes were examined to establish the efficacy of their protocol. Accordingly, all the tested aldehydes smoothly provided the products 100 and 101 in 91–97% and 91–96% yield, respectively, at 1.5–2 hours. The authors proposed a mechanism to recognize this reaction, which started with the initial Knoevenagel condensation between aldehydes 23 and malononitrile 96 under the influence of iodine to give the adduct Int-32 that can then experience nucleophilic addition from 98*via* Michael addition to form the intermediate Int-33. Treatment of Int-33 with NH_4_OH leads to the formation of intermediate Int-34. The intermediate Int-34 after cyclization forms 1,4-dihydropyridine Int-35 and yields the final product 100.

**Scheme 26:**
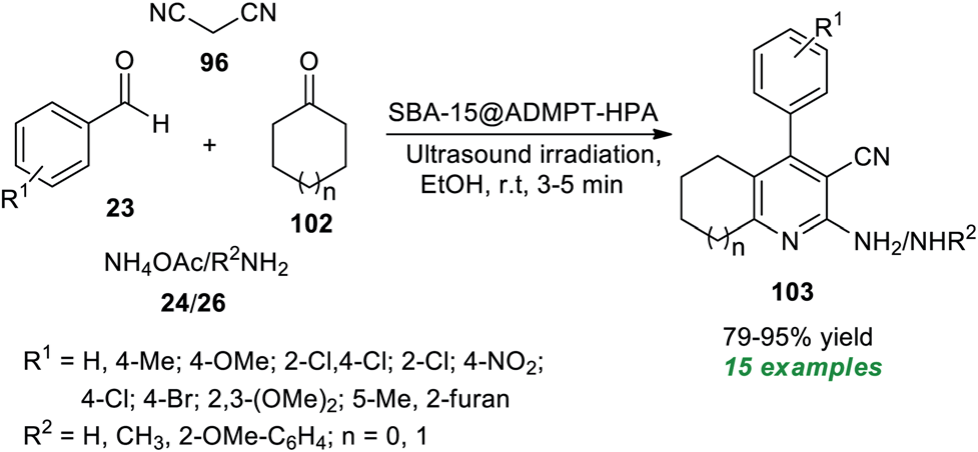
Sonochemical-assisted assembly of diverse pyridine derivatives.

At the same time, Dastjerdi and Ghanbari have prepared a novel nanohybrid catalyst *via* the functionalization of H_5_PW_10_V_2_O_40_ (HPA) with 2-APTS-4,6-bis(3,5-dimethyl-1*H*-pyrazol-1-yl)-1,3,5-triazine (ADMPT) linked SBA-15 mesoporous silica and the formation of the nanohybrid was entirely confirmed by FT-IR, TEM, SEM, BET, XRD, and EDX techniques (Scheme 26).^116^ The catalytic performance of the nanohybrid (SBA-15@ADMPT-HPA) was examined in the ultrasound irradiated one-pot reaction between aromatic/heteroaromatic aldehydes 23, with an amine source 24 or 26, malononitrile 96, and cyclic ketones 102 in ethanol as the solvent at room temperature. The catalyst was discovered to be very consistent in producing the respective pyridine products 103 and it could be separated easily and utilized for further consecutive cycles with a negligible loss in product yields. With the help of such an expeditious condition, a total of fifteen pyridine products 103 were synthesized in 79–95% yield in a relatively short time. The combination of the sonochemical activation strategy and the utilization of SBA-15@ADMPT/HPA as a heterogeneous catalyst efficiently makes this protocol eco-friendly and environmentally sustainable.

#### 3.1.2 Synthesis of quinolines

Recognizing the widespread appearance of quinolines and their analogues in the core skeleton of a variety of natural compounds and potential synthetic bioactive products, the efficient construction of this moiety has drawn immense interest in the area of medicinal and organic chemistry over the last few decades. Their versatile pharmacological application^117–119^ including antimalarial, antimicrobial, anticancer, antiprotozoal, anti-HIV, antitubercular, *etc.*, and materials science applications^120–123^ like photovoltaic cells, photographic plates, and OLEDs have sparked interest in the development of an efficient methodology for their successive construction and subsequent functionalization.^124–126^

**Scheme 27:**
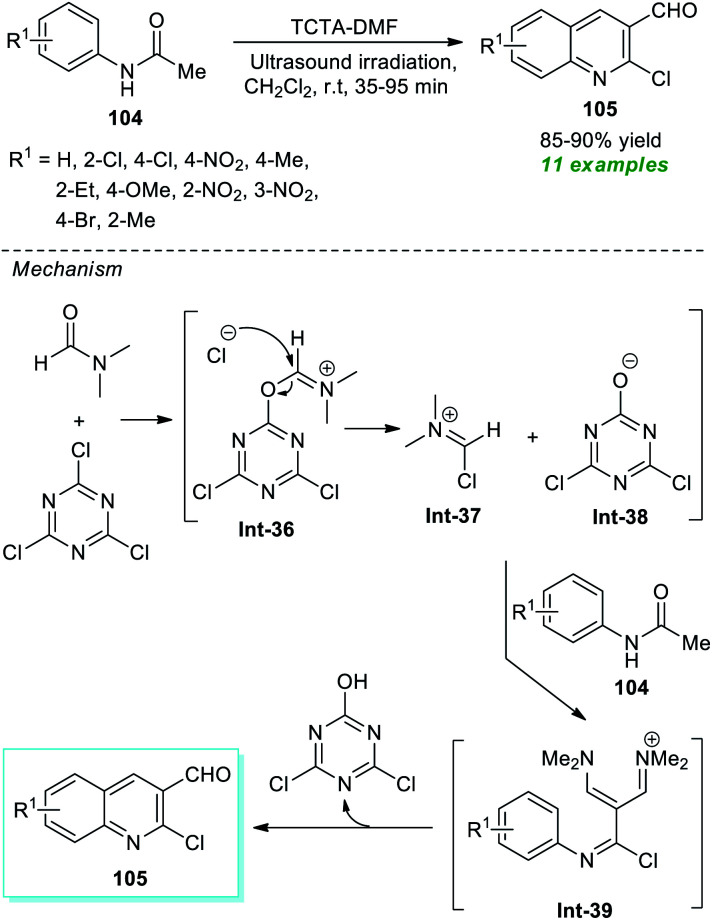
Expedient sonochemical synthesis of substituted quinolines.

The Rajanna group has demonstrated the synergic combination of 2,4,6-trichloro-1,3,5-triazine (TCTA) with *N*,*N*-dimethylformamide (DMF) as an effective Vilsmeier–Haack (VH) reagent for the ultrasound-assisted cyclization of acetanilides to form the substituted quinolines in high yield within 35–95 minutes (Scheme 27).^127^ This reaction condition was proven to be extremely successful in delivering eleven different compounds. Although the reaction could be accomplished using the traditional approach, it would take a considerably longer reaction time. Therefore, ultrasound irradiation was regarded as the method of choice for this reaction with respect to product derivation and environmental friendliness. Some of the appealing features of this protocol include the use of TCTA as an environmentally benign, easily accessible, and cost-effective substance, a simple reaction set-up, and broad substrate scope. The overall reaction can proceed through the formation of TCTA–DMF adduct Int-36 that could be transformed to chloromethyleniminum cation Int-37 for further reaction with acetanilides 104 to form the final products 105*via* intermediate Int-39.

**Scheme 28:**
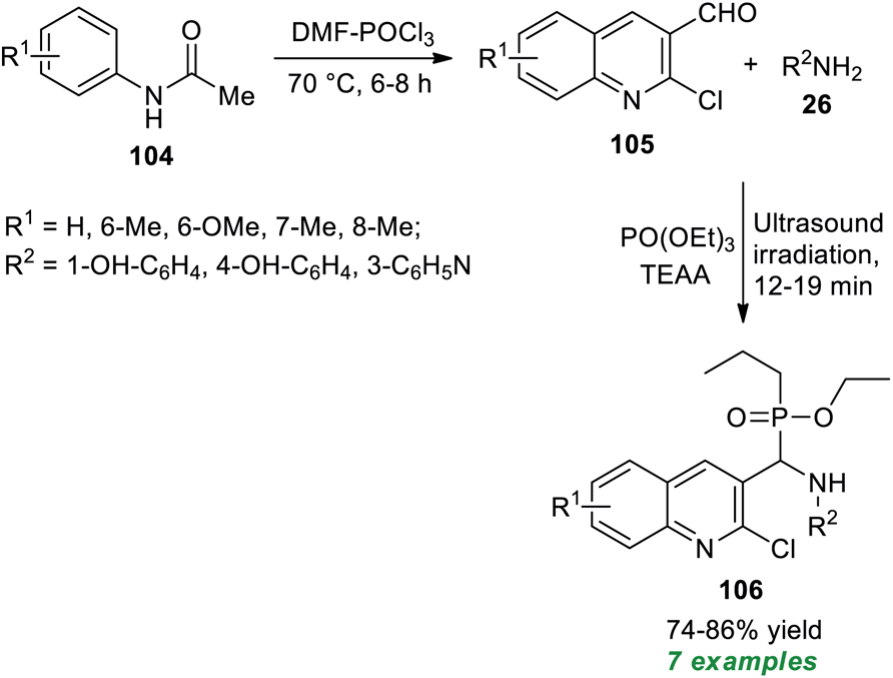
Ultrasound-assisted rapid access to quinolines bearing α-aminophosphonate.

Bazine *et al.*^128^ in 2020 synthesized a plethora of novel quinoline scaffolds bearing the α-aminophosphonate moiety *via* Kabachnik–Fields reaction by employing ionic liquid triethylammonium acetate (TEAA) as the catalyst and the solvent system under sonication (Scheme 28). The entire procedure begins with the formation of chloro-substituted formyl quinolines 105 from the condensation of acetanilides 104 with Vilsmeier–Haack reagent (DMF–POCl_3_) through the Meth–Cohn reaction.^129^ The so formed quinoline derivatives 105 on treatment with substituted amines 26 and PO(OEt)_3_ in the presence of TEAA in ultrasonication afforded the desired quinoline derivatives 106 bearing an α-aminophosphonate core in moderate to good yield. While executing the reaction without using TEEA, the rate of the reaction was found to be extremely slow, and the yield of the products was comparatively low. This protocol has many advantages, including a simple workup procedure, a fast completion rate, mild set-up, energy efficiency, and so on. However, the limited substrate scope and reasonably low product yields point toward the drawback of this method that necessitates future developments.

#### 3.1.3 Synthesis of pyrans

The development of a cost-effective green approach for designing and synthesizing bioactive heterocycles has remained a great challenge. Among various heterocycles, the 4*H*-pyran core and its derivatives are well-established heterocycles and commonly encountered in many more natural products, and synthetic drug type molecules.^130–134^ Several biologically active pyran heterocycles have been synthesized and established as antioxidant, fungicidal, antimicrobial, herbicidal, antitumor, and antiviral agents in the last decades.^135–138^ However, chemists' ever-increasing imagination of discovering new pathways for the synthesis of novel drug-type molecules by merging various pharmacological groups into single molecules for enhancing the properties of the parent molecules has still been a hot topic of current research.

**Scheme 29:**
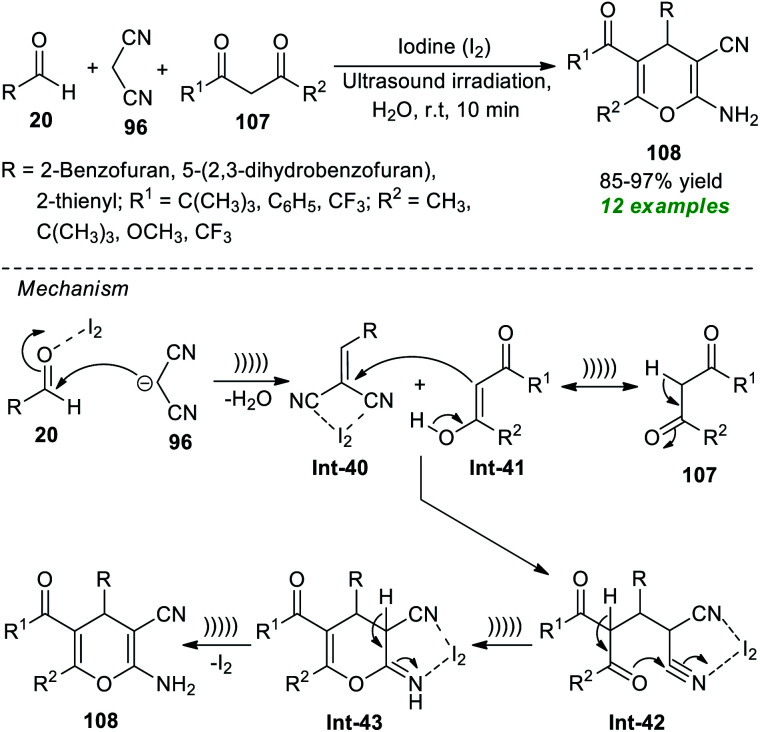
Iodine-catalyzed three-component sonochemical-assisted synthesis of 4*H*-pyrans.

To realize the importance of 4*H*-pyrans, particularly 2-amino-3-cyano-4*H*-pyrans, with the main focus on developing a green pathway, Pasha and co-workers have reported the utilization of iodine as a highly reactive and efficient catalyst in the sonochemical-assisted cyclo-condensation reaction of commercially accessible aldehydes 20, malononitrile 96, and substituted 2,3-diketones 107 in an aqueous medium to synthesize the respective 2-amino-3-cyano-4*H*-pyran products 108 only in 10 minutes (Scheme 29).^139^ The methodology was demonstrated to be feasible for a variety of 1,3-diketones and aldehydes, and a total of twelve target compounds 108 were accomplished in 85–97% yields. The utilization of ultrasound irradiation makes the protocol energy-efficient and reduces reaction times. The mechanism behind this reaction begins with the iodine-mediated Knoevenagel condensation of 20 and 96, thereby delivering the intermediate Int-40. In this step, the presence of iodine activates the carbonyl compounds towards the nucleophilic attack of malononitrile. Michael's addition of the enolate form Int-41 of 107 with the intermediate Int-40 delivers Int-42, which can then be cyclized to provide the intermediate Int-43. The consequent tautomerization of Int-43 yields the final products 108.

**Scheme 30:**
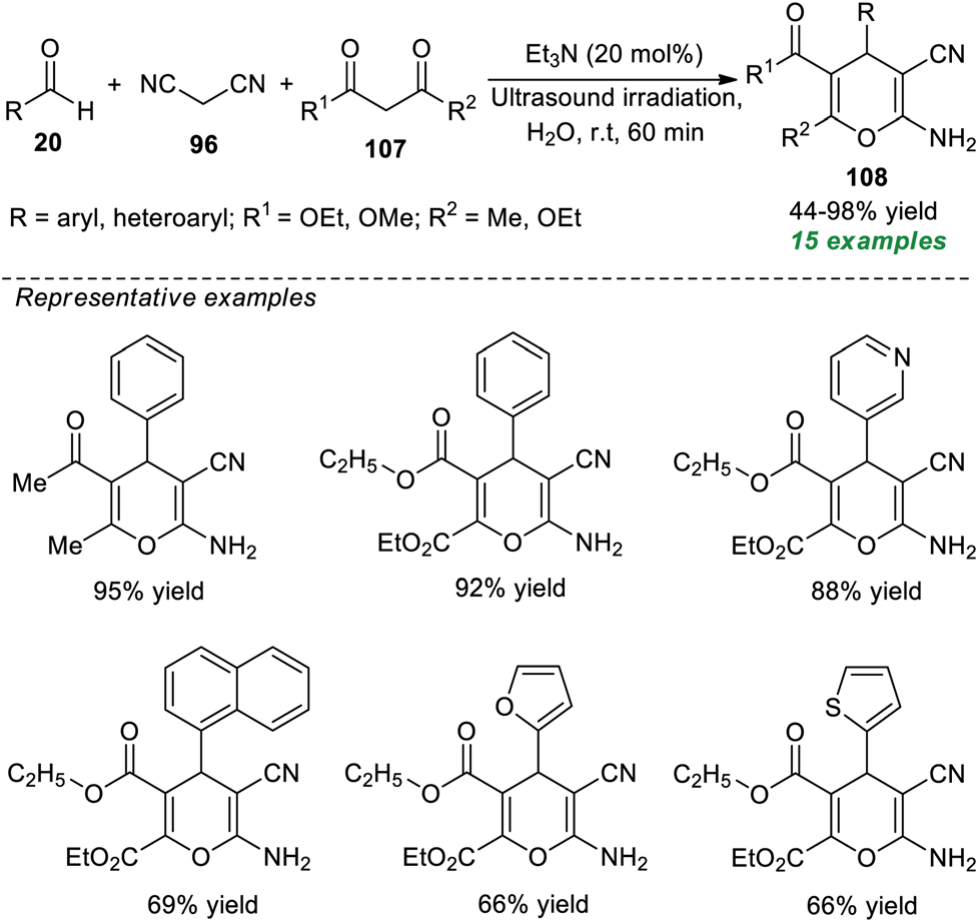
One-pot ultrasound-assisted construction of 4*H*-pyrans using Et_3_N as a catalyst.

Another successful application of ultrasound as an eco-friendly activation method to access 2-amino-3-cyano-4*H*-pyrans was demonstrated by the group of Herrera (Scheme 30).^140^ Under the influence of 20 mol% of Et_3_N as the organic base catalyst, an ultrasound-assisted three-component reaction of readily available aldehydes 20, malononitrile 96, and 1,3-diketones 107 was executed in water at room temperature for 60 minutes, which efficiently afforded the desired 4*H*-pyran products 108 in 44–98% yields. This operationally easy reaction worked well not only with aryl aldehydes but also with heteroaryl substituted aldehydes, resulting in a total of 15 compounds.

### 3.2 Synthesis of six-membered heterocycles containing two-heteroatoms

#### 3.2.1 Synthesis of pyrimidines

The six-membered two heteroatom containing nitrogen heterocycle pyrimidines and their derivatives have great potential in medicinal chemistry due to the diverse pharmacological activity of the target compounds, including antibacterial, antiplasmodial, anticancer, antineoplastic, anti-inflammatory activities, *etc.*^141–146^ Considering their wide chemical landscape and following the green chemistry principle, a diverse set of synthetic approaches have been devised.^147–149^

**Scheme 31:**
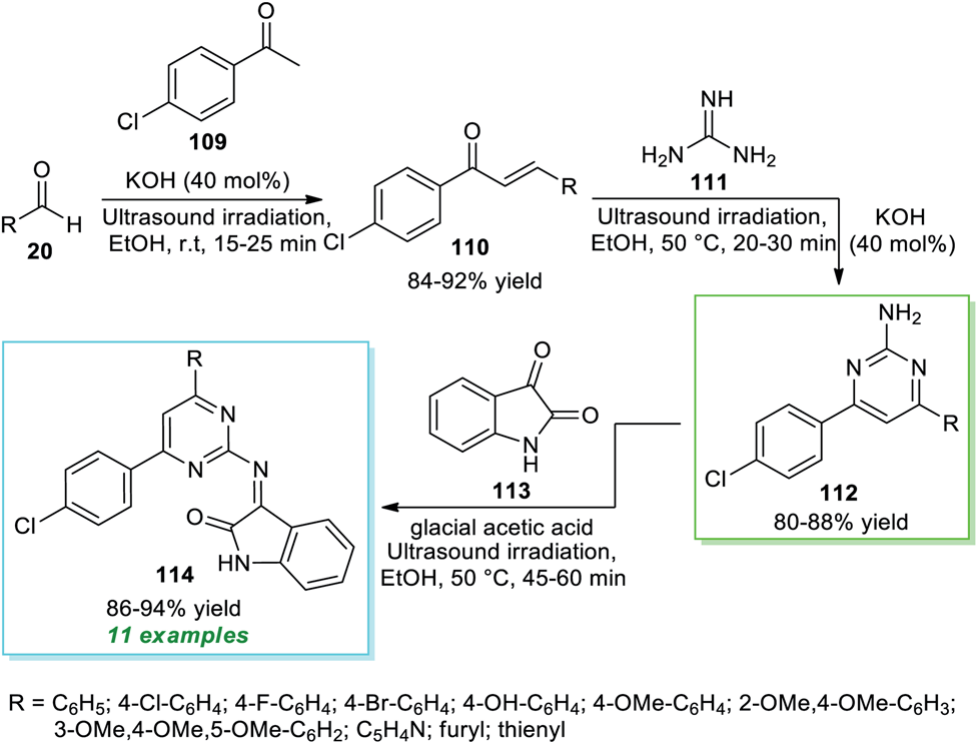
Ultrasound-assisted multistep synthesis of diversely substituted pyrimidines 114.

In 2018, Nikalje *et al.*^150^ disclosed an ultrasound irradiated step-wise strategy to realize the construction of 2-amino pyrimidine derivatives 114 coupled with indolin-2-ones (Scheme 31). Initially, the synthesis involves the ultrasound irradiation promoted condensation of 4-chloroacetophenone 109 with aryl/heteroaryl aldehydes 20 using 40 mol% of KOH as the base catalyst in EtOH at room temperature to form the enones 110 in 84–92% yield in 15–25 minutes. Although the reaction is achieved with conventional heating conditions, it takes a lot of time (4–6 hours) to deliver the products. In the next step, the ultrasound-assisted reaction of enones 110 and guanidine 111 was performed with the help of the same catalytic amount of KOH in ethanol at 50 °C for a time of 20–30 minutes that efficiently afforded the 2-amino substituted pyrimidine 112, which on further treatment with isatin 113 using glacial acetic acid as the catalyst in ethanol at 50 °C furnished the respective products 114 in 86–94% yield. Similarly, the formation of 112 and 114 from 110 can be done using thermal heating conditions; however, due to the requirements of a long reaction time in every step, sonochemical activation was recognized as the most effective approach in terms of reaction time as well as from the perspective of sustainable chemistry.

**Scheme 32:**
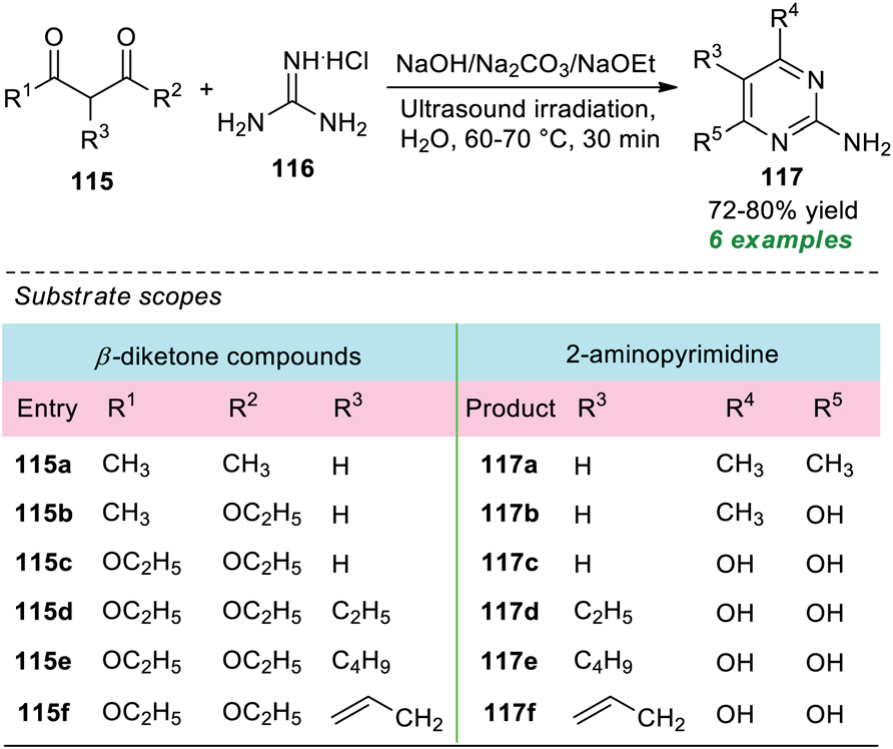
Base-catalyzed sonochemical synthesis of 2-aminopyrimidine in an aqueous medium.

A straightforward and energy-efficient green protocol for the construction of various 2-amino substituted pyrimidines was disclosed by Güllü and co-workers in 2019 (Scheme 32).^151^ Under ultrasound irradiation, the treatment of different β-diketone compounds 115 with guanidine hydrochloride 116 using various base catalysts including Na_2_CO_3_, NaOH, and NaOEt in an aqueous medium was found to smoothly proceed at 60–70 °C to deliver the corresponding 2-aminopyrimidine derivatives 117 in 72–80% yield. The presence of various substituents on the β-diketone ring plays a crucial role in this reaction as well as in the yield of the products. The β-diketone ring bearing ester moiety (R^1^ = OEt or R^2^ = OEt) underwent the reaction in the presence of NaOEt, and hydrolysis of the ester moiety has occurred, thereby delivering the 2-aminopyrimidine ring with an –OH group at 4- and 6-positions (R^4^ = R^5^ = OH), whereas methyl-substituted β-diketones (R^1^ or R^2^ = Me) were effectively worked under the influence of NaOH or Na_2_CO_3,_ and methyl-substituted 2-aminopyrimidine derivatives were observed. A comparison study of both conventional and ultrasound techniques revealed that a high-temperature condition of around 100 °C for a time of 5–6 hours was required when the reaction was carried out conventionally rather than sonochemically that was completed at only 60–70 °C in 30 minutes.

#### 3.2.2 Synthesis of quinoxalines

Quinoxalines and their derivatives, both of natural and synthetic origin, represent a broad class of nitrogen-heterocycles and have been regarded as one of the most privileged and prolific skeletons commonly existing in a wide range of natural products^152–155^ as potential therapeutic candidates,^156,157^ agrochemicals,^157,158^ and commercially available drugs.^159–161^ They have been established to exhibit a significant therapeutic potential such as antidiabetic, anticancer, anti-inflammatory, antimicrobial, anti-HIV, antituberculosis, antiviral, *etc.*^162^ Because of the signs mentioned above of quinoxalines, a wide range of synthetic protocols have been devised for their construction.^162^

**Scheme 33:**
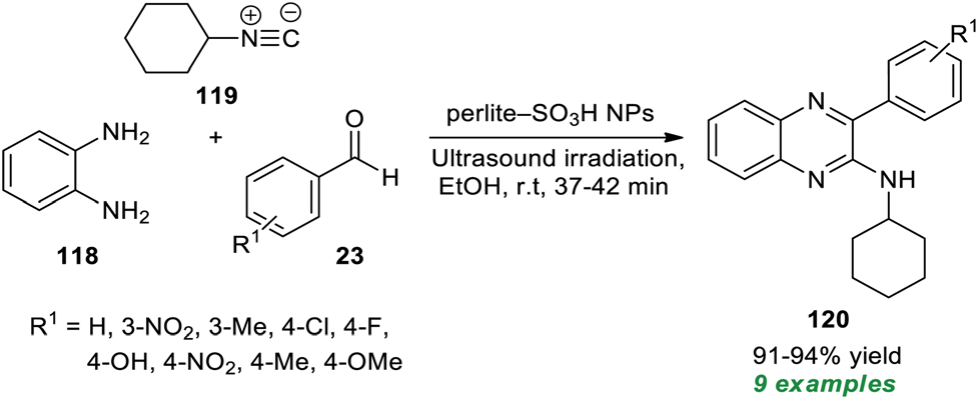
One-pot three-component sonochemical synthesis of quinoxalines.

To realize the outstanding participation of quinoxalines in drug design and discovery, Rouhani and Ramazani in 2018 disclosed an isocyanide-based multicomponent approach for rapid access to a variety of highly functionalized quinoxalines by introducing ultrasound irradiation as a robust alternative activation approach (Scheme 33).^163^ In this regard, a one-pot three-component reaction between aldehydes 23, with 1,2-diamine 118, and isocyanide 119 in the presence of perlite-SO_3_H nanoparticles as the efficient catalyst was performed in ethanol under ultrasound irradiation for a time of 37–42 minutes at room temperature. The reaction condition tolerates a wide variety of aryl aldehydes with different electron-poor, and electron-rich substituents, and a total of nine quinoxaline products 120 were synthesized in 91–94% yield. Although the preferred quinoxaline products could be synthesized using the thermal method, the reaction rate was found to be exceedingly sluggish, and the yield was not satisfactory, which recommended the sonochemical approach as the method of consideration from the viewpoint of green and synthetic chemistry.

**Scheme 34:**
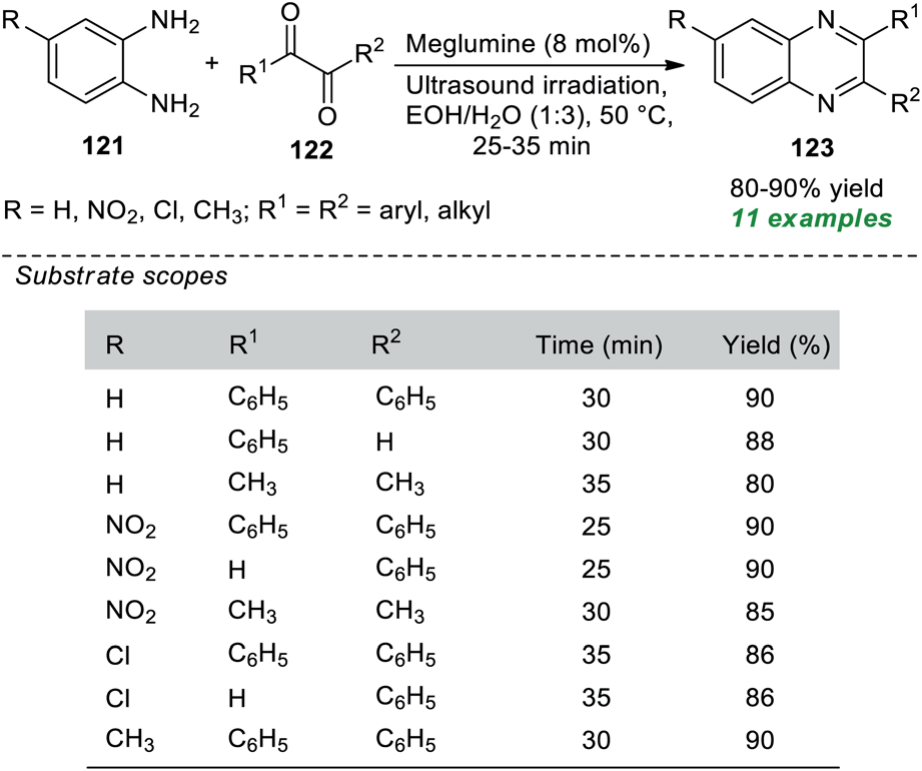
Organocatalytic ultrasound-assisted two-component synthesis of quinoxalines.

Nongkhlaw *et al.* in 2019 pioneered the use of meglumine as a biodegradable organocatalyst in association with the application of sonochemical activation for the production of various quinoxaline derivatives 123 (Scheme 34).^164^ Initial optimization of the appropriate amount of the catalytic system and reaction medium for the reaction of 1,2-diamine 121 with 1,2-diketone 122 revealed that only 8 mol% of the catalyst was sufficient for catalyzing this reaction and aqueous ethanol was found to be the best solvent under sonication as well as under conventional heating conditions. However, the reaction requires much more time under traditional heating conditions, and the desired quinoxaline products have been achieved in low yield in contrast to the reaction executed under ultrasound irradiation. A variety of *o*-phenylenediamines, as well as aryl and alkyl-substituted 1,2-diketone, were well tolerated by this mild approach, and a total of eleven quinoxalines were obtained in 80–90% yield. Broad functional group tolerance, operational simplicity, cost-effectiveness, being environmentally benign, and toxic-free are some of the advantages of this protocol.

#### 3.2.3 Synthesis of quinazolinones

The bicyclic heterocycles, possessing a pyrimidine ring fused at five- and six-positions with a benzene moiety, commonly known as quinazolinones, belonging to a promising class of nitrogen-heterocycles, are ubiquitously distributed in the core structure of numerous natural products^165–167^ and medicinally privileged compounds.^168,169^ Over the last decades, the design and development of efficient methods for the construction of this compound received ample attention^170–173^ for their potential therapeutic applications, including antimicrobial, anti-HIV, antidepressant, anticancer, antidiabetic, anti-inflammatory, and analgesic.^174–177^

**Scheme 35:**
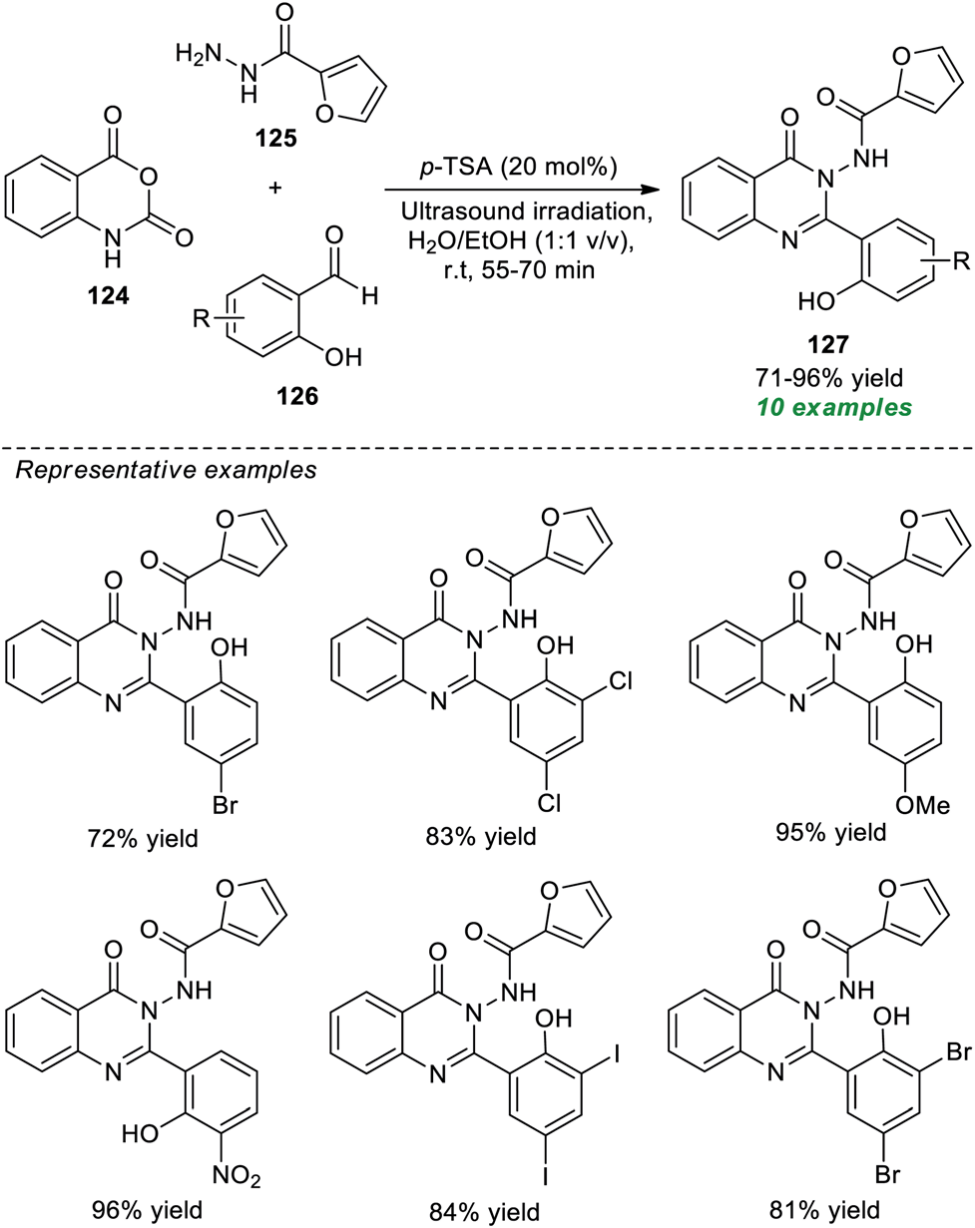
Acid-catalyzed multicomponent synthesis of quinazolinones under sonication.

Given the properties mentioned above, Latip, Seo, and co-workers devised an atom-economical and practical one-pot multicomponent strategy to access a plethora of highly functionalized quinazolinones under ultrasound irradiation conditions (Scheme 35).^178^ With the help of 20 mol% of *p*-TSA as the catalytic system, the desired quinazolinone products 127, derived from the reaction of isatoic anhydride 124, 2-furoic hydrazides 125, and various substituted salicylaldehydes 126 in aqueous ethanolic solution under ultrasound irradiation at ambient temperature for 55–70 minutes, were obtained in 71–96% yields. The salicylaldehydes with various electron-rich and electron-poor groups have seemed to be efficiently worked under the standard conditions and have no negative impact on the yield of the products. Some of the prepared compounds were also identified as inhibitors of the tyrosinase enzyme. The utilization of sound waves as an efficient green method, mixed green solvents, short reaction time, and easy isolation process are some of the key highlights of this approach.

**Scheme 36:**
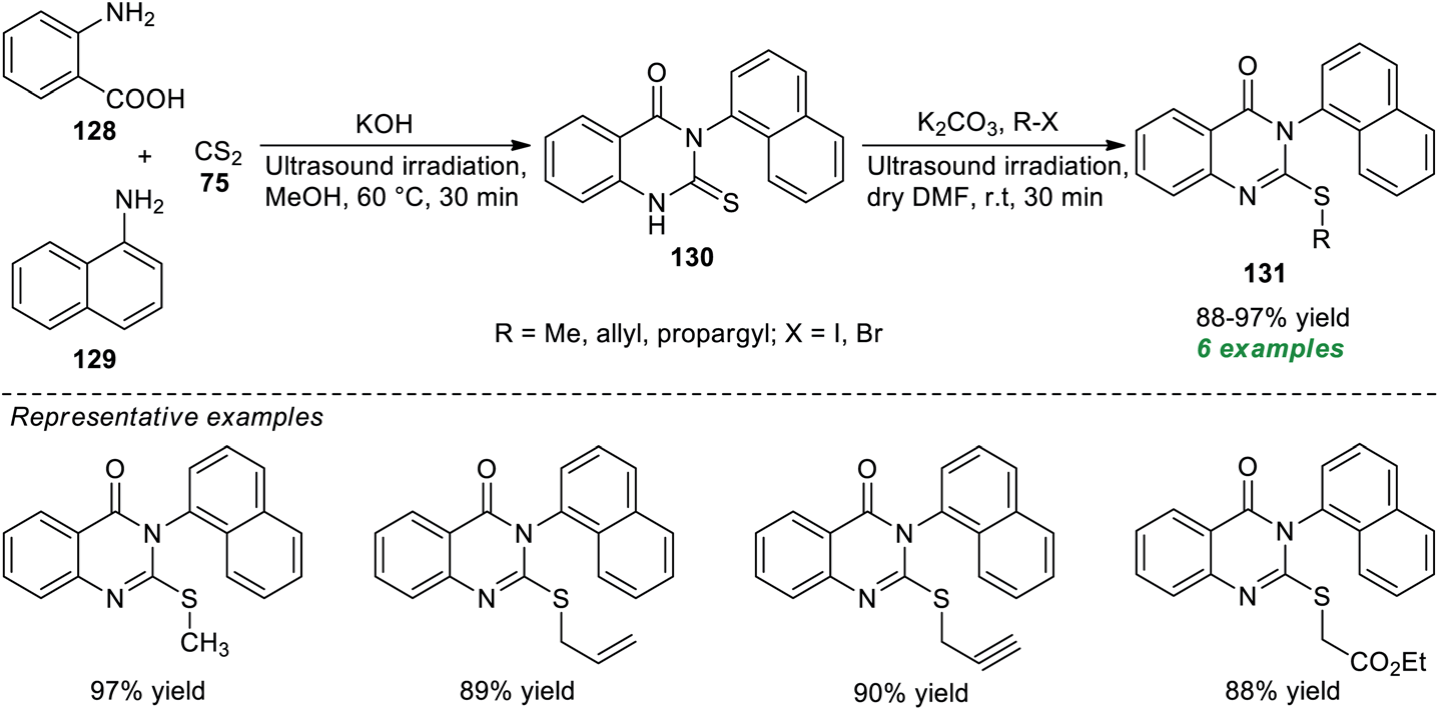
Ultrasound-assisted multicomponent reaction mediated two-step synthesis of quinazolinones.

A facile and straightforward protocol for expedient access to a variety of alkyl-substituted quinazolinones has been disclosed by Hagar, Ashry, and their group in 2021 (Scheme 36).^179^ With the help of KOH as a base catalyst, an ultrasound-assisted treatment of anthranilic acid 128, 1-naphthylamine 129, and carbon disulfide 75 was found to occur smoothly at 60 °C to lead to the products 130, which on further reaction with different alkyl halides catalyzed by K_2_CO_3_ in dry DMF under ultrasound irradiation at room temperature furnished the corresponding products 131 in 88–97% yields. Although the present methodology was categorized as efficient, eco-compatible, and sustainable, and the products were easily obtained without using column chromatography, the limited number of substrates denotes a shortcoming of this procedure, demanding further advancements in broadening the substrate scope, otherwise outstanding developments.

## 4. Ultrasound irradiation-promoted transition-metal-free synthesis of complex fused poly-heterocycles

A broad spectrum of complex fused heterocyclic compounds in transition-metal-free catalysis under ultrasound irradiation have been achieved in the last few years. The synthesis of these compounds where several heterocycles are combined in such a way that leads to the formation of new single molecules from easily available simple starting materials has recently received considerable attention from chemists and pharmacologists for the outstanding properties of the targeted compounds.^180–182^

**Scheme 37:**
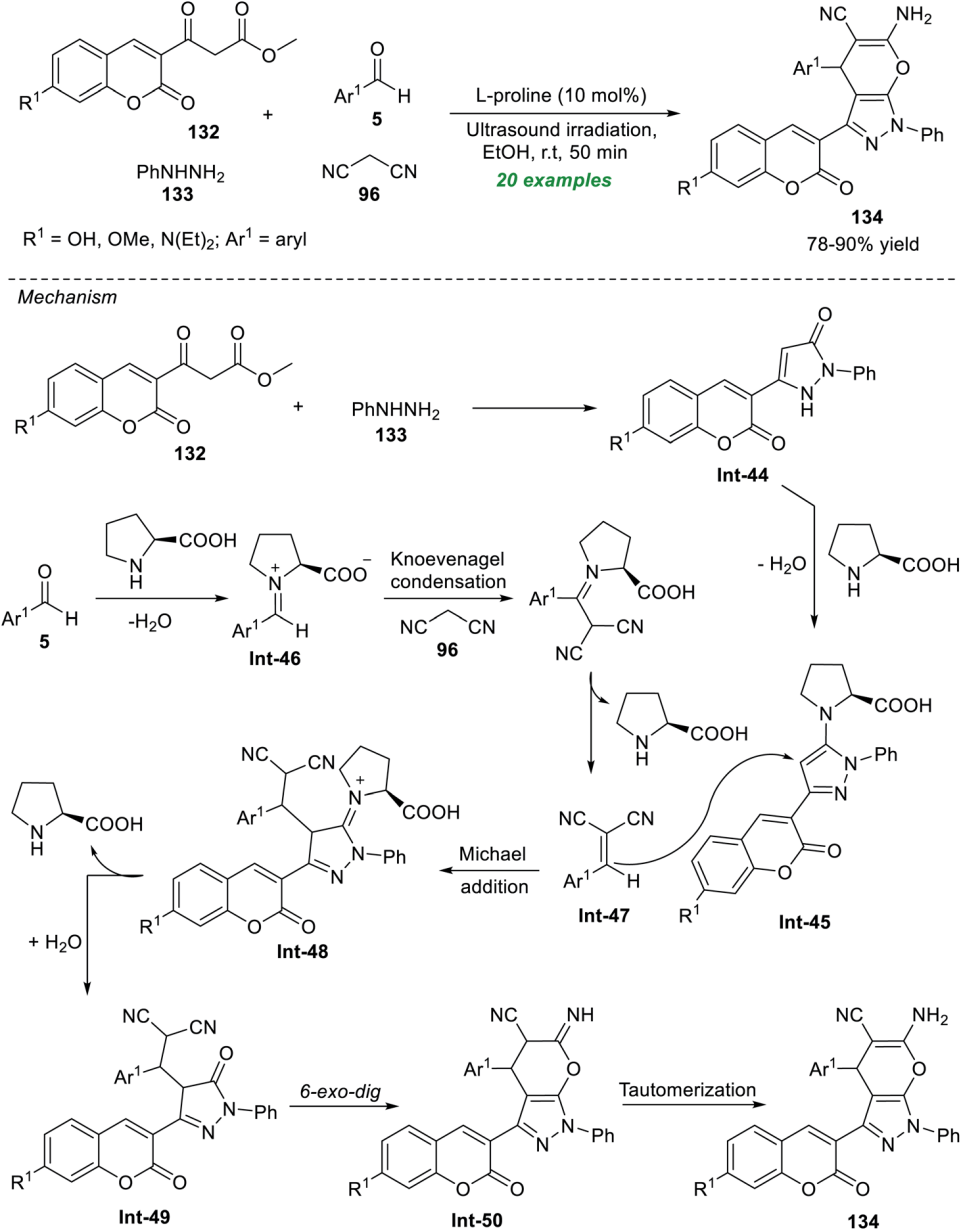
Organocatalytic sonochemical synthesis of pyrano[2,3-*c*]pyrazoles linked with coumarin.

The development in this regard realizes the outstanding contribution of Seydimemet *et al.* to the expedient synthesis of a plethora of coumarin-containing pyrano[2,3-*c*]pyrazoles by introducing the sonochemical activation strategy under organocatalytic conditions (Scheme 37).^183^ Under the influence of 10 mol% of l-proline, the one-pot domino reaction of β-ketoesters 132 with phenylhydrazine 133, aromatic aldehydes 5, and active methylene 96 in ethanol was carried out in sonication, which provided the respective products 134 in 78–90% yield after 50 minutes. Broad functional group tolerance, efficiency, fast reaction rate, cost-effectiveness, and toxic-free nature are some highlights of this methodology. The formation of 134 can be initiated *via* the initial reaction of 132 with 133 to produce the coumarin–pyrazolone intermediate Int-44, which afforded the intermediate Int-45 under the influence of l-proline. In the meantime, l-proline catalyzed condensation of 5 with 96 yields the intermediate Int-47. The subsequent conjugate addition of Int-45 to Int-47 afforded the intermediate Int-49. The final 6-*exo-dig* cyclization of intermediate Int-49 and then tautomerization *via* intermediate Int-50 yields the actual product 134.

**Scheme 38:**
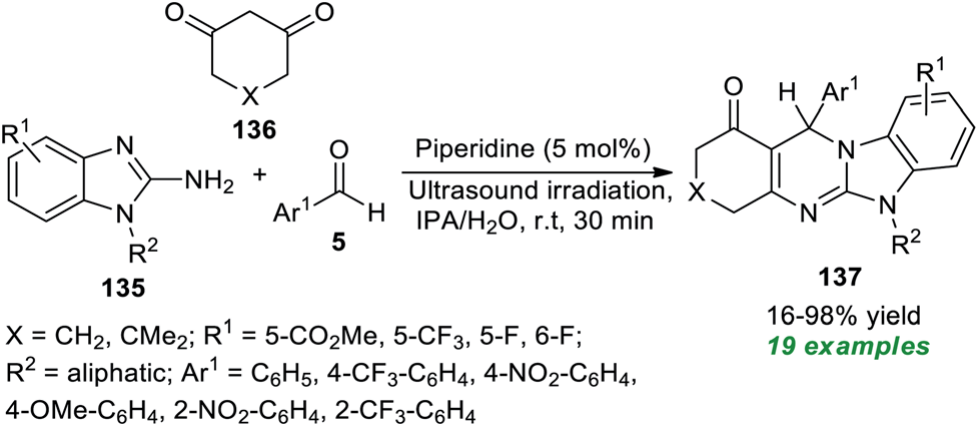
Three-component organocatalyzed synthesis of benzimidazo[2,1-*b*]quinazolin-1(1*H*)-ones under sonication.

Sun *et al.*^184^ disclosed an ultrasound-irradiated three-component reaction of 2-amino-benzimidazoles 135, aryl aldehydes 5, and 1,3-dione 136 by employing 5 mol% of organic base piperidine as the catalyst for the assembly of diversely substituted imidazo[2,1-*b*]quinazolinones 137 (Scheme 38). During the optimization of the reaction parameters, a variety of solvents were tested, including CH_2_Cl_2_, MeOH, THF, toluene, CH_3_CN, DMF, IPA, H_2_O, and IPA/H_2_O; among them, IPA/H_2_O turned out to be an efficient solvent system for this reaction, delivering the products in excellent yield in a short duration of time. A diverse set of aromatic aldehydes with varied electron-poor and electron-rich substituents were examined to broaden the substrate scope, and all of them have been discovered to proceed efficiently under optimal conditions. Similarly, substitution on the 2-amino-benzimidazole ring and 1,3-diketone was found to have no substantial effect on the yield of the products, and all have smoothly participated in the reaction. A total of nineteen benzimidazo[2,1-*b*]quinazolin-1(1*H*)-one products 137 were synthesized in poor to excellent yield. This procedure has a broad spectrum of substrate scope, is less time-consuming, and is cost-effective. The comparatively low yield of the product 137 (R^1^ = 5-CO_2_Me, R^2^ = cyclohexane, X = CH_2_, Ar^1^ = 4-OMe-C_6_H_4_) somewhat denotes a limitation of this approach. This is most likely because of the presence of the 4-OMe group on the aldehyde ring, which leads to the formation of 4-methoxybenzyl alcohol and 4-methoxy benzoic acid as by-products.

**Scheme 39:**
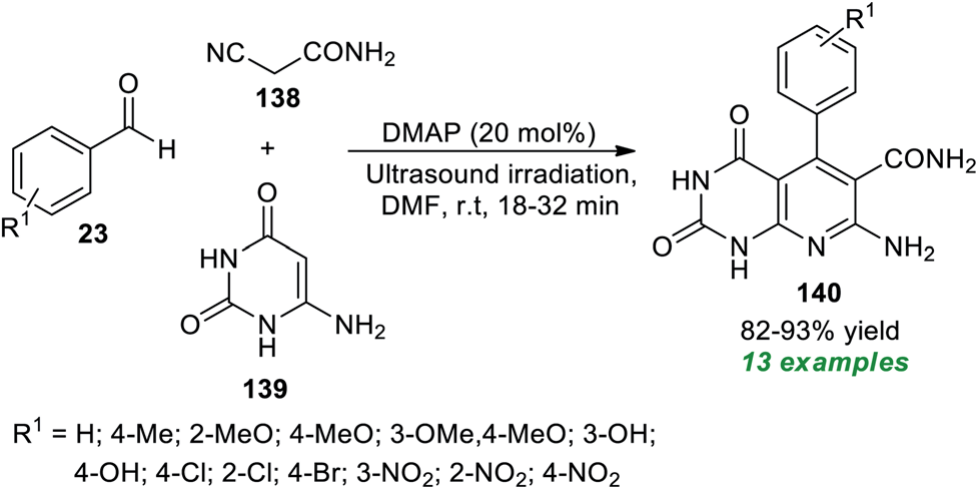
DMAP-catalyzed ultrasound-assisted synthesis of pyrido[2,3-*d*]pyrimidines 140.

Construction of pyrimidine fused bioactive compounds, namely pyrido[2,3-*d*]pyrimidine 140 through a domino Knoevenagel–Michael addition initiated multicomponent reaction under ultrasound conditions has been achieved by Bhat and co-authors (Scheme 39).^185^ By employing 20 mol% of DMAP (4-dimethylaminopyridine) as an organocatalyst, the corresponding products 140, accomplished from the three-component reaction of readily available aldehydes 23, active methylene compounds 138, and 6-aminouracil 139 in DMF at room temperature, were obtained in 82–93% yields. The reaction was initially performed under various catalysts such as DMAP, DBU, DABCO, piperidine, and morpholine in different solvent systems like H_2_O, MeOH, EtOH, CH_2_Cl_2_ and DMF. Among the catalysts and solvents tested, the utilization of DMAP as the catalytic system and DMF as the solvent system was found to be highly efficient for the fast reaction rate and for providing a good yield of the products. The optimal condition was extremely compatible for a variety of aromatic aldehydes containing electron-rich and electron-poor substituents on the different positions of the aromatic ring of the aldehydes. When compared to the traditional thermal method, which took nearly 2–6 hours, ultrasound irradiation reduces the reaction time from 32 minutes to 18 minutes. Similarly, when applying ultrasound techniques, product yields were boosted by up to 93%, compared to the conventional method, which only provides 67–89% yields.

Considering their wide-ranging chemical landscape and biological properties, pyrazolone moieties were recognized as a versatile building block for the creation of diverse heterocyclic compounds. Among them, dihydropyrano[2,3-*c*]pyrazole is a well established fused heterocycle synthesized from pyrazolone, which displays various pharmacological properties like anti-bacterial, anti-HIV, insecticidal, anti-infective, anti-platelet, anti-fungal, anti-cancer, anti-microbial, antioxidant, analgesic activity, *etc.*^79,181,186,187^

**Scheme 40:**
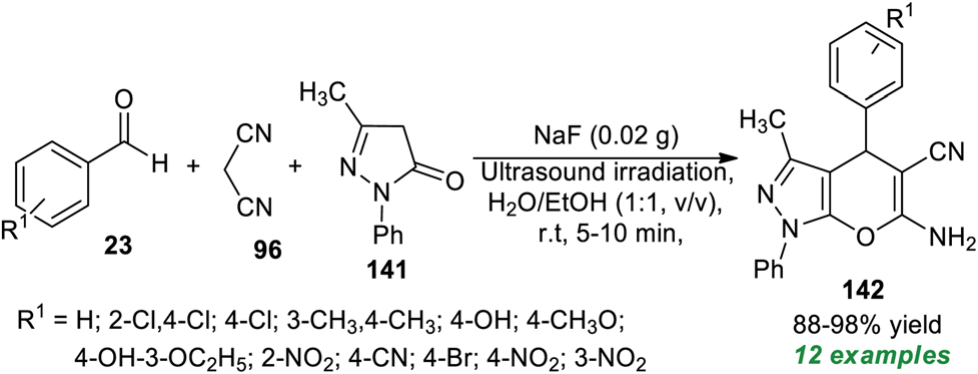
NaF-catalyzed MCRs to access pyrano[2,3-*c*]pyrazoles under sonication.

Due to the above mentioned pivotal significance of pyrano[2,3-*c*]pyrazoles, Kotha and his co-workers demonstrated an atom-economic approach for the rapid access to a library of pyrano[2,3-*c*]pyrazoles 142 from a three-component ultrasound-assisted reaction of aldehydes 23, malononitrile 96, and pyrazolone 141 in aqueous ethanolic solution (1 : 1, v/v) at room temperature using sodium fluoride (NaF) as the base catalyst (Scheme 40).^188^ A diverse range of aryl aldehydes bearing different substituents were efficiently worked by this method, and overall, 12 target compounds have been accomplished in 88–98% yield within only 5–10 minutes. The broad functional group tolerance, reduced reaction time, cost-effectiveness, clean reaction profile, low catalyst loading, and green solvents mark the highlights of this protocol.

**Scheme 41:**
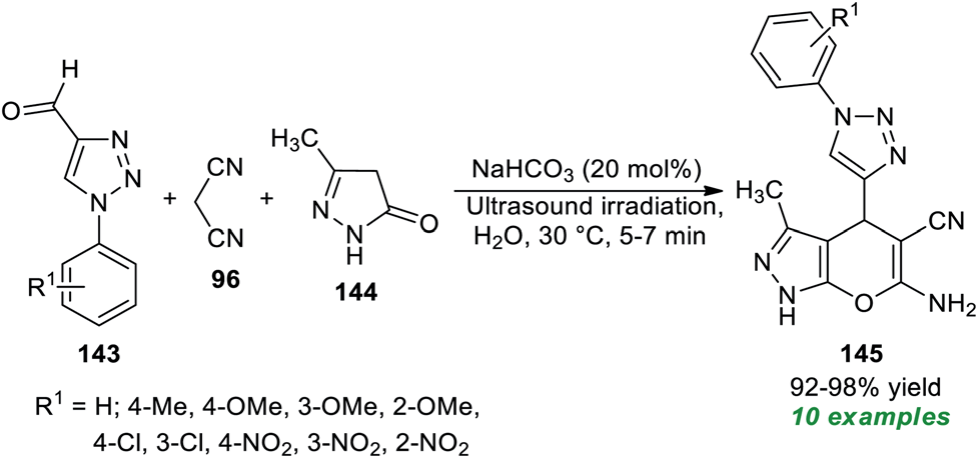
NaHCO_3_-catalyzed sonochemical synthesis of triazole-based pyrano[2,3-*c*]pyrazoles.

Subsequent to this report, a three-component tandem reaction of triazolyl aldehydes 143, malononitrile 96, and pyrazolone 144 was carried out in ultrasound irradiation by employing 20 mol% of NaHCO_3_ as the base catalyst in water at 30 °C for 5–7 minutes (Scheme 41).^189^ This reaction afforded the 1,2,3-triazolyl based pyrano[2,3-*c*]pyrazoles 145 in 92–98% yields. Although different mild bases such as Na_2_CO_3_ and K_2_CO_3_ could tolerate the reaction, it took a lot of time, and excellent yield was achieved only when NaHCO_3_ was used rather than Na_2_CO_3_ or K_2_CO_3_. The authors evaluated the potential therapeutic properties of the synthesized compounds, and most of the compounds were found to be efficient anti-fungal and antioxidant agents.

**Scheme 42:**
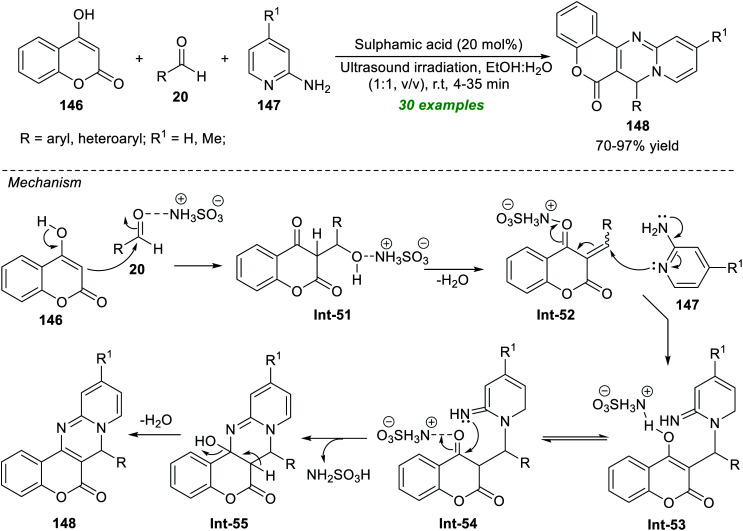
Sulfamic acid-catalyzed multicomponent synthesis of chromeno[4,3-*d*]pyrido[1,2-*a*]pyrimidin-6(7*H*)-ones under sonication.

Brahmachari *et al.*^190^ established a simple, straightforward, and environmentally benign multicomponent approach for the practical construction of structurally divergent chromeno[4,3-*d*]pyrido[1,2-*a*]pyrimidin-6(7*H*)-ones (Scheme 42). By introducing 20 mol% of sulphamic acid (NH_2_SO_3_H) as an organocatalyst, they executed an atom- and pot-economic reaction between readily accessible 4-hydroxycoumarin 146, substituted aldehydes 20, and 2-aminopyridine 147 with an equimolar mixture of aqueous ethanolic solution (1 : 1, v/v) under ultrasound irradiation under ambient conditions, which provided the respective products 148 in 70–97% yields. Both aromatic aldehydes featuring diverse substitution on C-3 and C-4 positions and substituted heteroaromatic aldehydes reveal this reaction condition to be entirely compatible. The use of sonochemistry in this reaction shortens the reaction time and results in a clean, high-yield synthesis of the products. It's worth noting that no co-catalyst, additives, or reagents are required for the reaction. The broad substrate scope, clean and green solvents, high reaction mass efficiency, and high atom efficiency mark the highlights of this protocol. The overall transformation starts with the initial NH_2_SO_3_H catalyzed Claisen–Schmidt condensation reaction between 20 and 146, producing 52. The subsequent nucleophilic addition of Int-52 with 147 delivers the adduct Int-53 that can then undergo tautomerization and cyclization to afford the intermediate Int-55. Finally, the elimination of water from the intermediate Int-55 yields the corresponding product 148.

**Scheme 43:**
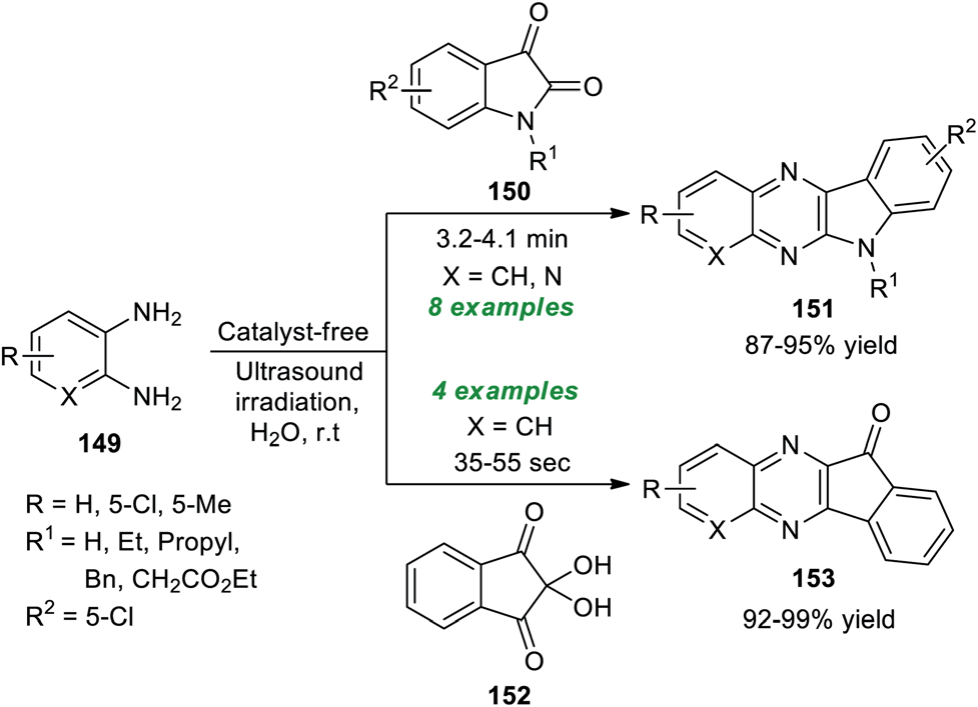
Ultrasound-assisted synthesis of diverse fused quinoxalines.

A simple and highly convenient strategy to synthesize a diverse set of fused quinoxaline scaffolds under ultrasound irradiation from readily available *o*-phenylenediamine was devised by Srivastava *et al.* (Scheme 43).^191^ The synthesis involves the treatment of *o*-phenylenediamine 149 with isatin 13 under the catalyst-free conditions in the presence of water as the green reaction medium in sonication at room temperature, which afforded the desired complex-fused quinoxaline products, namely indolo[2,3-*b*]quinoxalines 151 in good to excellent yields within 3.2–4.1 minutes. Similarly, under the standard experimental parameter, the treatment of 149 with ninhydrin 152 yielded a variety of indeno[1,2-*b*]quinoxalines 153 in outstanding yields in a short interval of time. This reaction effectively proceeded for a range of diverse substitutions on the isatin ring and the 1,2-diamine ring. Both unsubstituted isatins (R^1^ = H) and substituted isatins (R^1^ = Et, Bn, and Pr) reacted efficiently under the standard reaction conditions.

**Scheme 44:**
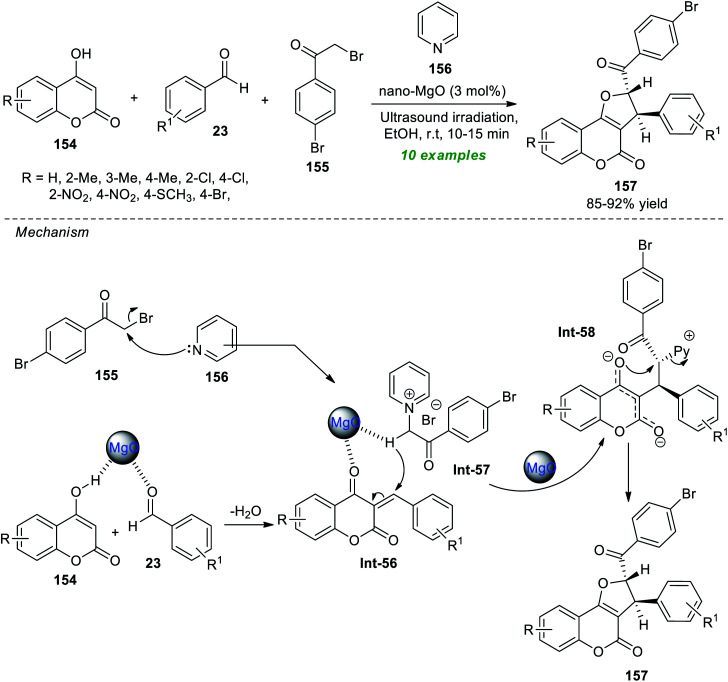
MgO-catalyzed ultrasound-assisted synthesis of furo[3,2-*c*]coumarin.

Safaei-Ghomi *et al.*^192^ demonstrated the ultrasound irradiation technique as an eco-friendly activation method for the highly diastereoselective synthesis of coumarin-fused furans, namely furo[3,2-*c*]coumarins 157 (Scheme 44). Under the influence of 3 mol% of magnesium oxide nanoparticles (MgO NPs) as a highly reactive catalyst, the respective products 157 were accomplished from readily available substituted 4-hydroxycoumarin 154, aromatic aldehydes 23, bromo-substituted acetophenone 155, and pyridine 156 in ethanol at room temperature. Overall ten compounds have been synthesized by this reaction as a single diastereomer in an 85–92% yield. The reaction was very fast and completed within only 10–15 minutes under sonication compared to the conventional heating condition, which marks the advantage of this approach. The synthetic potentiality of the present method was established from the successful gram-scale synthesis of the model compounds with reduced catalyst loading. Other notable features of this technique include broad substrate scope, simple work-up procedures, easy product isolation with no tedious column chromatography techniques, easy separation of the catalyst, and re-utilization for further reactions with negligible loss in catalytic activity. On the other hand, the catalyst necessitates multistep synthesis at significantly higher temperatures, which indicates the drawback of this method. The postulated mechanism for realizing this reaction involves the MgO promoted Knoevenagel condensation of 154 and 23, thereby producing the intermediate Int-56 on the surface of the catalyst. The pyridinium ylide Int-57 is produced from the reaction of 155 and 156, and then undergoes Michael addition with Int-56. The final product 157 was obtained *via* intramolecular cyclization of the resultant intermediate Int-58, along with the elimination of pyridines.

**Scheme 45:**
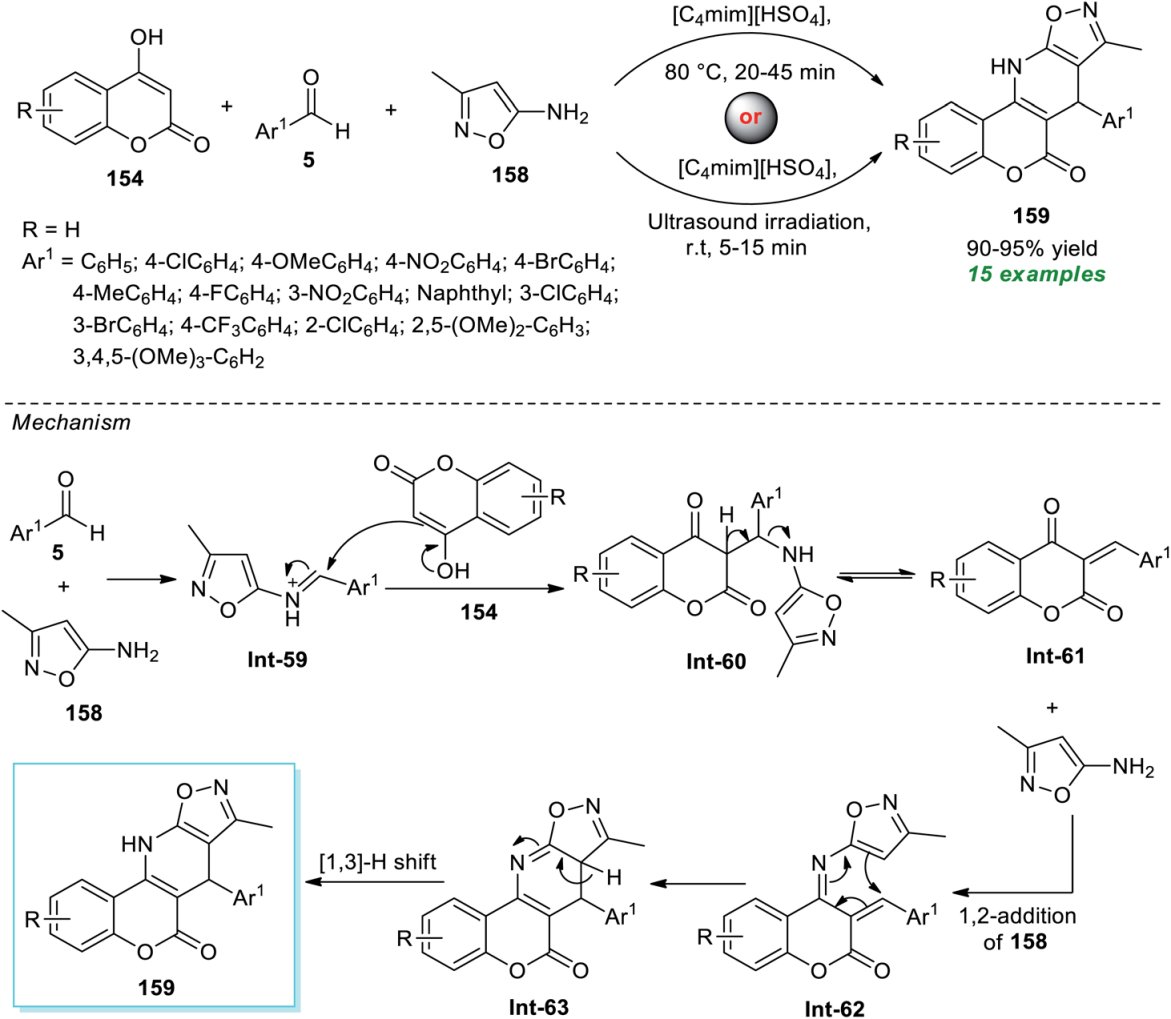
Sonochemical access to chromeno[3,4-*e*]isoxazolo[5,4-*b*]pyridin-6-one using ionic liquid catalysis.

Kumari and co-workers illustrated an eco-compatible and sustainable protocol by demonstrating the prolific activity of the ionic liquid as a solvent as well as catalytic system towards the synthesis of a library of dihydro-6*H*-chromeno[3,4-*e*]isoxazolo[5,4-*b*]pyridin-6-ones 159 (Scheme 45).^193^ Under the influence of [C_4_mim][HSO_4_] as the ionic liquid, both traditional thermal methods and ultrasound techniques were used to execute a three-component reaction between 4-hydroxycoumarin 154, aryl/heteroaryl aldehydes 5, and amino-substituted isoxazoles 158. From the experimental outcome, it was revealed that the reaction conducted with conventional heating at 80 °C took almost 20–45 minutes to yield the corresponding products 159 in 90–95% yield, whereas the same reaction when irradiated with ultrasound at room temperature took only 5–15 minutes to produce the expected product 159 in nearly the same quantity. Even though both of these parameters were proven to be effective, the ultrasound technique surpassed the conventional one with respect to reaction time and energy consumption. A total of 15 compounds bearing different substituents on the aryl- and heteroaryl rings of the aldehydes were synthesized by this method. In this regard, the authors proposed a mechanism that starts with the initial condensation of aldehydes 5 with amino-isoxazole 158 to afford the intermediate Int-59 that experiences nucleophilic attack from 4-hydroxycoumarin 154, thereby offering intermediate Int-61. The subsequent 1,2-addition of Int-61 with 158 followed by cyclization leads to the formation of intermediate Int-63. Consequently, a [1,3]-H shift of Int-62 provides the corresponding product 159.

**Scheme 46:**
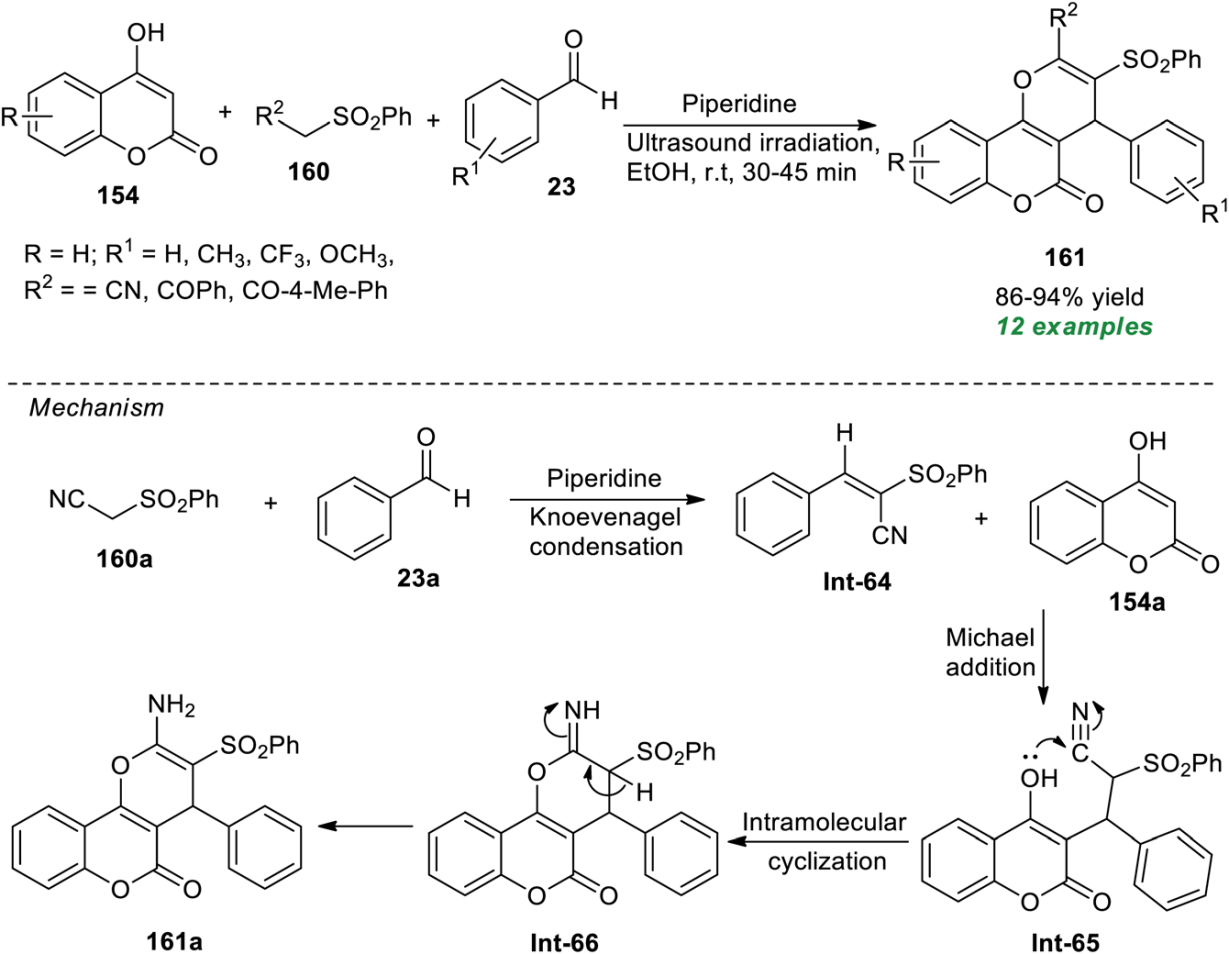
Piperidine-catalyzed ultrasound-assisted multicomponent assembly of pyrano[3,2-*c*]coumarin.

Construction of a diverse set of pyrano[3,2-*c*]coumarin derivatives 161 has been accomplished by Al-bogami *et al.*^194^ from the piperidine catalyzed one-pot atom-economical reaction of substituted benzaldehyde 23, methylene compounds 160, and highly reactive 4-hydroxycoumarin 154 under ultrasound irradiation (Scheme 46). The reaction was performed at room temperature with ethanol as the best optimum solvent. Noticeably, all reactants were found to efficiently participate in the reaction in 30–45 minutes, and the target compounds 161 were achieved in 86–94% yields. To broaden the substrate scope, a variety of aromatic aldehydes with varied electron-donating and electron-withdrawing groups and various substitutions on methylene compounds were employed. All are suitably worked under the optimized reaction condition. Although it is possible to execute the reaction using the conventional method to synthesize the desired products, due to the required long reaction time and high energy input, it has limitations from the perspective of synthetic potentiality and sustainability. The sonochemical approach sped up the reaction and increased the product yield, and the entire process took only a few minutes. To explain this transformation, the authors proposed a mechanism that begins with piperidine catalyzed Knoevenagel condensation of active methylene compounds 160a with aldehydes 23a. The resulting intermediate Int-64 undergoes Michael addition with 4-hydroxycoumarin 154a to generate the intermediate Int-65. The final product 161a is obtained *via* intramolecular cyclization of Int-65 and concurrent tautomerization of resultant intermediate Int-66.

**Scheme 47:**
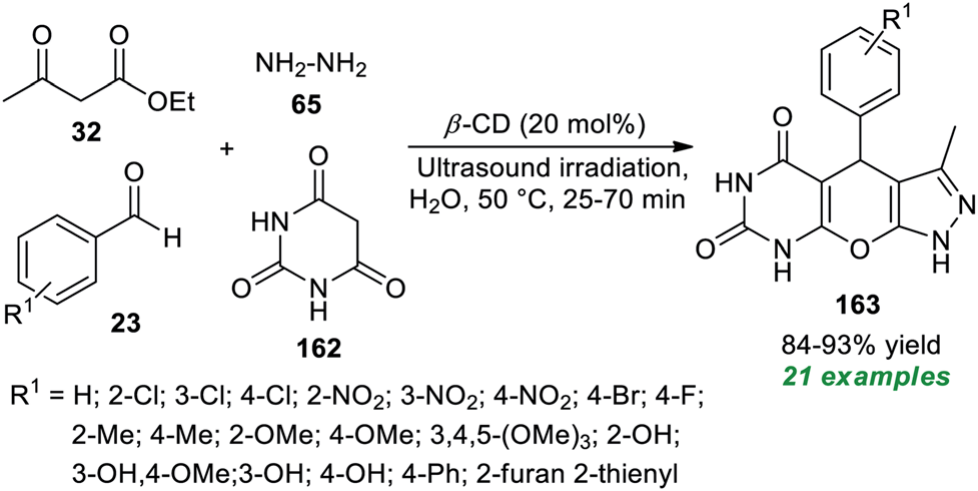
Supramolecular catalysis in the sonochemical synthesis of pyrazolo-pyrano[2,3-*d*]pyrimidine.

Application of supramolecular catalysis in the ultrasound-assisted expedient construction of pyrazolo-pyrano[2,3-*d*]pyrimidine framework 163 has been demonstrated by Shingate and co-workers in 2020 (Scheme 47).^195^ In this regard, a one-pot reaction of aldehydes 23, barbituric acid 162, ethyl acetoacetate 32, and hydrazine 65 in an aqueous medium was performed with the help of 20 mol% of β-cyclodextrin as a supramolecular catalyst under sonication at 50 °C for 25–70 minutes. This four-component reaction furnished the corresponding products 163 in 84–93% yields. This approach was successful in synthesizing a total of 21 molecules possessing various electron-poor and electron-rich groups on the aromatic as well as the heteroaromatic ring of aldehydes. The feasibility of the current approach was validated by proving the catalyst's recyclability and reusability for the next successive reaction sequences without altering the significant outcome of the protocol.

**Scheme 48:**
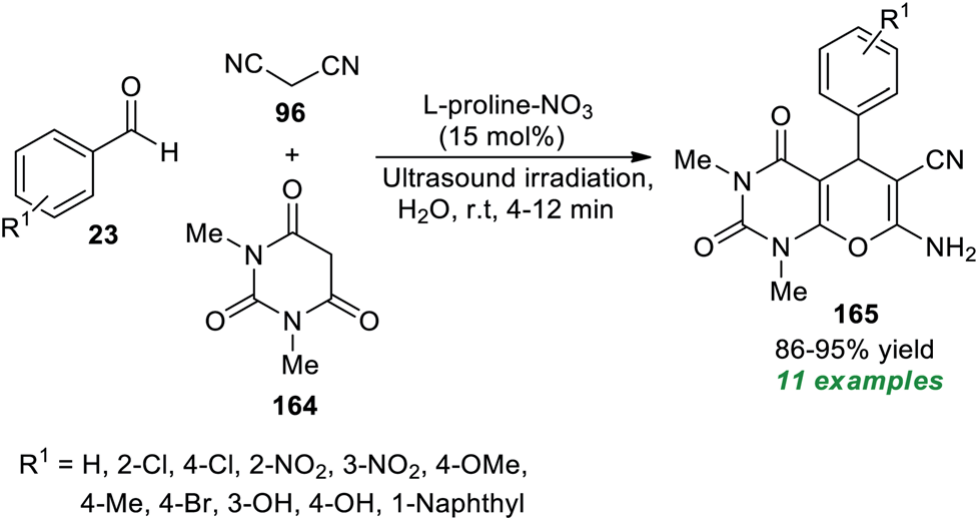
l-Proline-NO_3_-catalyzed three-component synthesis of pyrano[2,3-*d*]pyrimidine under sonication.

Another great achievement for the utilization of ultrasound irradiation in the construction of complex-fused heterocycles by introducing l-proline-based ionic liquids as the efficient homogeneous catalyst was disclosed by More *et al.* at the same time (Scheme 48).^196^ Initially, the ionic liquid l-proline-NO_3_ was prepared by the authors from the reaction of l-proline with HNO_3_ in water at 60 °C for 24 hours. After the successful formation of the catalyst, a three-component reaction between aryl/heteroaryl aldehydes 23, malononitrile 96, and 1,3-dimethyl barbituric acid 164 in water was executed using both traditional heating and ultrasonication to examine the catalytic activity of the prepared catalyst. Only 15 mol% of the catalyst was found to be sufficient for catalyzing the reaction, as per the observations. This reaction yielded the corresponding pyrano[2,3-*d*]pyrimidine products 165 in 86–95% yields in 4–12 minutes with ultrasound irradiation at room temperature. In contrast, the reaction under conventional heating conditions (80 °C) produced the respective products in a lower yield than the ultrasound method.

## 5. Ultrasound-assisted synthesis of complex spiro-heterocycles under transition-metal-free conditions

Spiro heterocycles are considered the privileged molecular framework commonly encountered in the basic skeleton of a diverse range of naturally occurring molecules and synthetic pharmaceutical compounds.^197–200^ A vast array of compounds possessing a spirocyclic core in their structure are known for their antitumor, antimicrobial, antibiotic, antitubercular, and anticancer activities.

**Scheme 49:**
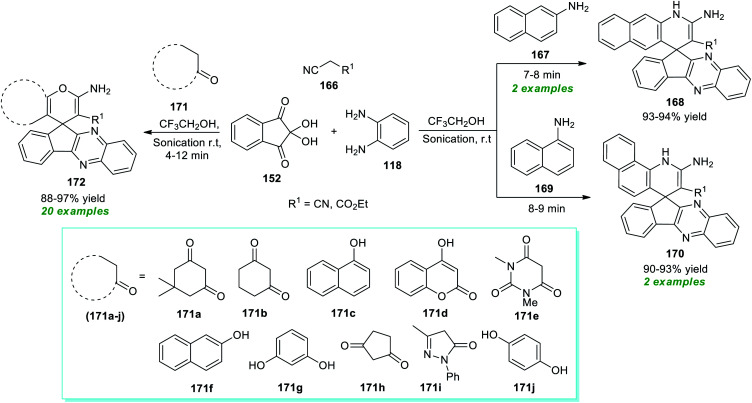
Construction of a library of spiro-heterocycles under ultrasound irradiation.

In this perspective, Heravi and Norouzy successfully demonstrated the use of ultrasound irradiation in the construction of a library of spiro-heterocycles *via* a multi-component approach by employing catalyst-free conditions (Scheme 49).^201^ Using trifluoroethanol (TFE) as the reaction medium, the four-component reaction between readily accessible 2,2-dihydroxy-1*H*-indene-1,3(2*H*)-dione 152, benzene-1,2-diamine 118, active methylene compound 166, and naphthalen-2-amine 167 or naphthalen-1-amine 169 yielded the corresponding spiro-benzoquinoline-indenoquinoxaline 168 and spiro-benzoquinoline-indenoquinoxaline 170 in 93–94% and 90–93% yield at 7–9 minutes respectively. Interestingly, replacing the amines with various CH-activated acidic compounds 171 was found to efficiently react with 2,2-dihydroxy-1*H*-indene-1,3(2*H*)-dione 152, benzene-1,2-diamine 118, and active methylene compound 166 under the same reaction condition, and a series of diverse highly functionalized fused pyran substituted spiro-indeno[2,3-*b*]quinoxaline products 172 were accomplished in 88–97% yield within 4–12 minutes. The extraordinary demands of relatively high energy conditions along with the prolonged timeframe associated with the conventional method for the completion of the reaction as compared to ultrasound irradiation methods that take place only in a short duration of time make the ultrasound method best for the synthesis of these compounds from the synthetic as well as green chemistry point of view.

**Scheme 50:**
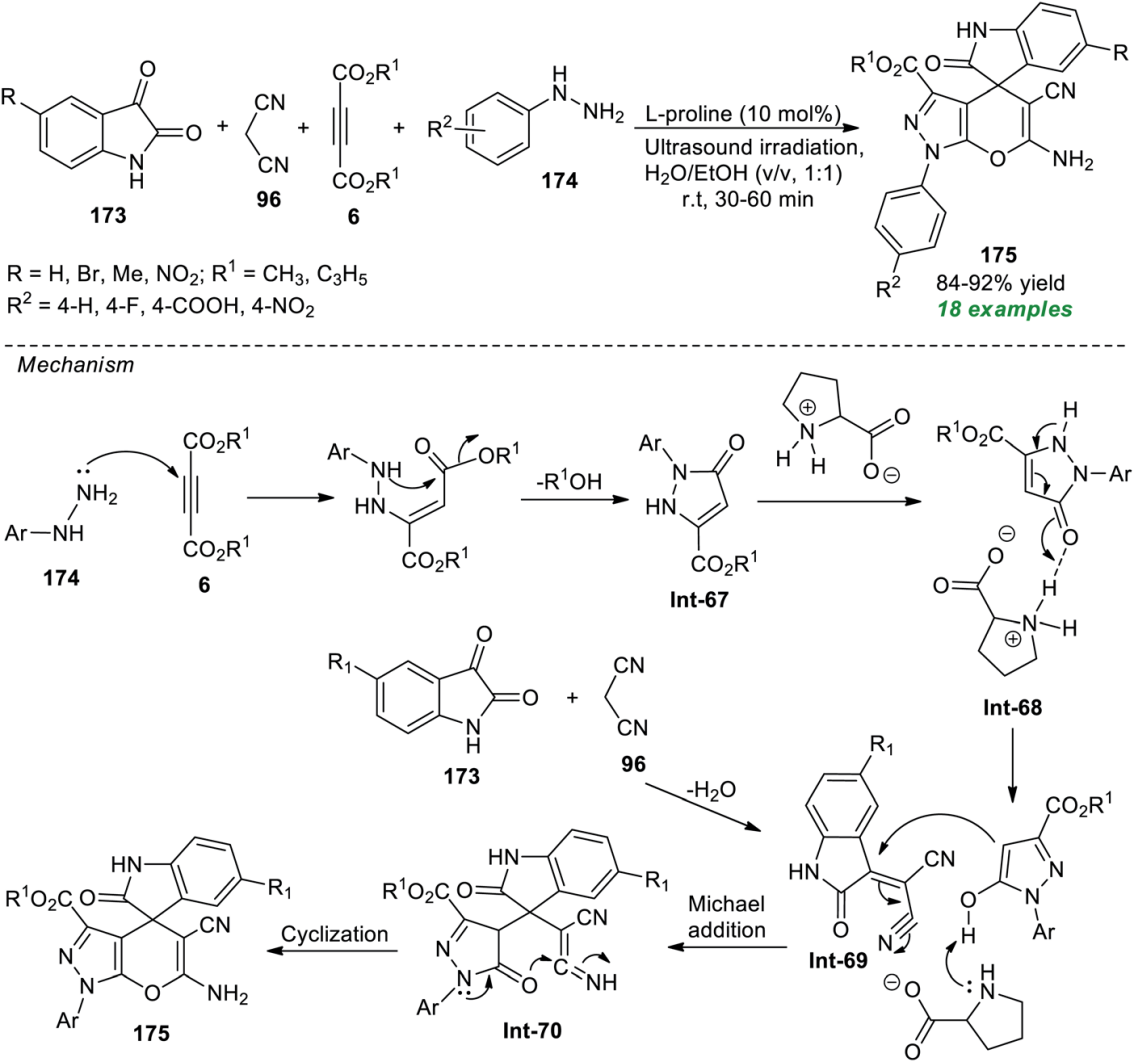
Secondary amine-catalyzed one-pot sonochemical synthesis of amino-substituted spiro-pyrano[2,3-*c*]pyrazoles.

A rapid, and straightforward one-pot ultrasound-assisted secondary amine l-proline catalyzed approach for the synthesis of spiro[indoline-3,4-pyrano[2,3-*c*]pyrazoles] was devised by Liju and co-workers from the four-component reaction of various isatins 173, malononitrile 96, dialkyl acetylene dicarboxylates 6, and phenyl substituted hydrazines 174 in aqueous ethanolic solution at room temperature (Scheme 50).^202^ This reaction required a very low loading of the catalyst and following this condition, a diverse range of target compounds 175 have been synthesized in 84–92% yield within 30–60 minutes. The methodology was found to be compatible only with *N*-unsubstituted isatin. Therefore, there is a need for extending the protocol to *N*-substituted isatin. To explain the possible formation routes to 175, the authors proposed a mechanism that is depicted in Scheme 50. Initially, an exothermic reaction of 174 with 6 can take place to form pyrazolone derivatives Int-67 which then produce the l-proline activates enolate intermediate Int-68. The Michael addition of the resulting –OH form of pyrazolone with Int-69 (produced from the reaction between 173 and 96) yields the intermediate Int-70. The subsequent cyclization of intermediate Int-70, as well as tautomerization, furnished the corresponding products 175.

**Scheme 51:**
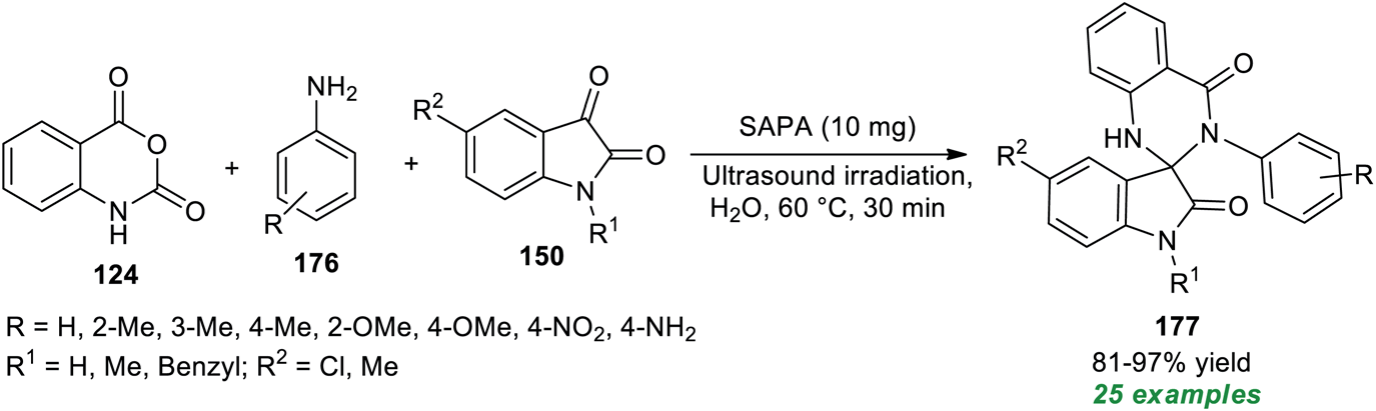
Ultrasound-assisted acid-catalyzed construction of spiro-oxindole-based quinazolines.

As illustrated in Scheme 51, treatment of isatoic anhydride 124, various arylamines 176, and isatins 150 in water under the influence of a phytic acid-based solid catalyst (SAPA) in sonication at 60 °C afforded a plethora of spiro-oxindole embedded dihydro-quinazolinones 177.^203^ The catalyst was found to be very efficient for this reaction and could be recycled and reused for further successive reaction sequences without altering the synthetic potentiality of the protocol. This reaction offers a total of 25 compounds in 81–97% yield within only 30 minutes. Variations in the substitution of different groups on the aryl ring of amines had no substantial effect on the reaction rate or product yield. Both *N*-substituted and *N*-unsubstituted isatins were found to be quite compatible with this approach.

**Scheme 52:**
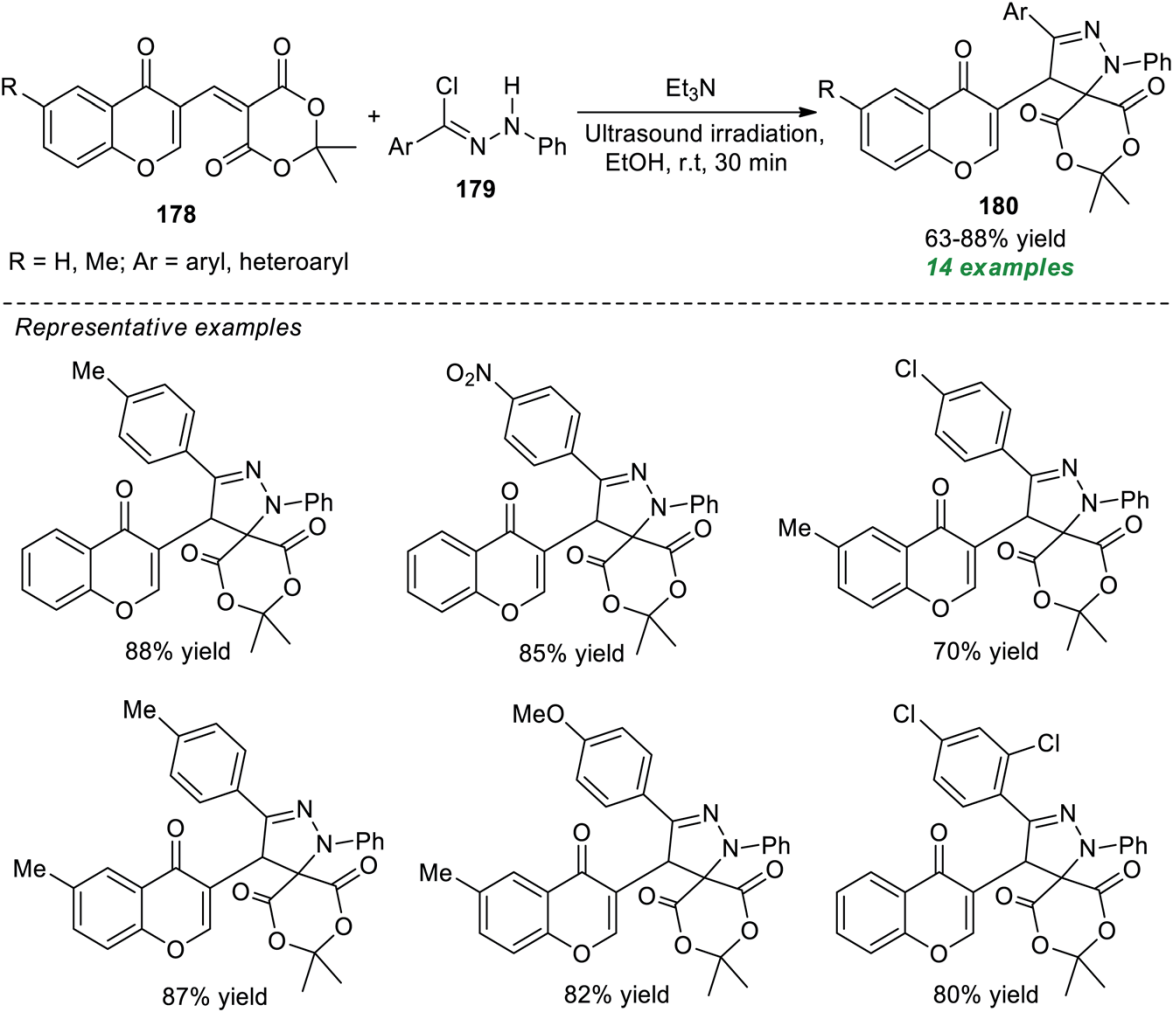
Reverse 1,3-dipole-mediated [3 + 2]cycloaddition for the synthesis of spiropyrazolines under sonication.

An eco-compatible and facile constructive approach for expedient access to a variety of spiropyrazoline derivatives 180*via* reverse 1,3-dipole mediated [3 + 2]cycloaddition of 2,2-dimethyl-5-[(4-oxo-4*H*-chromen-3-yl)methylene]-1,3-dioxane-4,6-dione 178 with nitrile imines produced *in situ* from hydrazonoyl chlorides 179 in the presence of Et_3_N as the catalyst under ultrasound irradiation was developed by Yavari and Fadakar (Scheme 52).^204^ The authors screened different types of catalytic systems including DIPEA, K_2_CO_3_, Et_3_N, Cs_2_CO_3_, DBU, and DABCO, and solvent systems such as EtOH, CH_2_Cl_2_, DMF, EtOAc, and H_2_O at room temperature for this reaction and identified that Et_3_N in EtOH under sonication at room temperature was the best combination for this reaction.

## 6. Conclusion and future perspective

Recognizing the frequent occurrences of heterocyclic compounds in the basic skeleton of diverse ranges of natural products, potential bioactive compounds, pharmaceutical agents, and optoelectronic materials, the development of efficient methodologies to realize their synthesis as well as for converting easily accessible raw materials into significant structural scaffolds that find huge application in many branches of chemistry has remained a formidable challenge for chemists and pharmacologists. Nevertheless, the people of this millennium are fully conscious of the importance of protecting their living environment from pollution caused by various hazardous reagents and solvents in the form of chemical waste during a chemical process both on industrial and laboratory scales.

In this pursuit, the field of synthetic organic chemistry has witnessed outstanding growth in the last decades for modifying synthetic chemical processes with the main focus to reduce the cost-effectiveness of the transformation by introducing environmentally benign conditions to offer a hazard-free and sustainable environment. Accordingly, the emergence of ultrasound irradiation as an alternative efficient activation method has opened up new frontiers for carrying out diverse organic transformations. It has a lot of advantages over traditional heating conditions, being eco-friendly and consuming less amount of energy. Consequently, the development of potential synthetic routes that can be operated in sonication is becoming extremely significant both scientifically and technically. In particular, sonochemistry in transition-metal-free catalysis offers a lot of opportunities in the recent efforts to establish green and more sustainable chemistry. The successful combination of the features of ultrasound irradiation as an environmentally acceptable activation method and the efficiency of transition-metal-free catalysis in promoting chemical reactions is predominantly relevant in achieving the ambition of green and sustainable chemistry.

Concerning this prominent significance, the present review article aims to highlight the recent progress accomplished in the application of ultrasound irradiation as a non-conventional energy source for the synthesis of a wide variety of oxygen, nitrogen, and sulfur-containing five-membered and six-membered heterocycles as well as complex-fused heterocycles and spiro-heterocycles by employing transition-metal free catalysis. We endeavored to highlight the drawbacks and limitations of the existing protocols in addition to demonstrating the successful improvements made in the creation of diverse heterocycles using these ecologically friendly approaches. The possible scope of future developments is also highlighted.

From the aforementioned observation made in this review, it is clear to conclude that the appearance of sonochemistry in transition-metal-free catalysis allows for all the reactions to be carried out in the absence of high energy conditions, toxic and expensive metal catalysts, or co-catalysts, ligands, volatile organic solvents or hazardous reagents. The use of water, ionic liquid, deep eutectic solvents, and other solvents such as ethanol, PEG, and aqueous ethanol makes the reported work environmentally and eco-friendly benign. In addition, solvent-free methods are being developed in metal-free environments using ultrasound irradiation. Furthermore, the majority of the synthesized compounds were discovered to have significant therapeutic activities. Notwithstanding these developments, some of the previously existing techniques have drawbacks such as narrow substrate scope, low product yields, and high catalyst loading. Consequently, considerable attention still needs to be paid to improving and expanding the scope of the reactions, with an outstanding product selectivity, by introducing readily accessible raw materials that are easy to handle and cost-effective and developing a scalable protocol with ultralow catalyst loading that will find a wider application to the industrial area.

We hope the information presented here will help researchers for identifying the current evolution accomplished in the synthesis of diverse-heterocyclic scaffolds under sonication under metal-free conditions as well as in developing many more precise and concise efficient synthetic routes using various metal-free catalysts especially organocatalysts, and stimulating further inventive developments that could find immense application to many branches of chemistry.

## Conflicts of interest

There are no conflicts to declare.

## Supplementary Material
